# Mucous Permeable Nanoparticle for Inducing Cuproptosis‐Like Death In Broad‐Spectrum Bacteria for Nebulized Treatment of Acute Pneumonia

**DOI:** 10.1002/advs.202408580

**Published:** 2025-02-22

**Authors:** Huiqun Hu, Shiyuan Hua, Feng Lu, Wenting Zhang, Zengwen Zhang, Jiarong Cui, Xiaoyue Lei, Jingyan Xia, Feng Xu, Min Zhou

**Affiliations:** ^1^ Department of Infectious Diseases The Second Affiliated Hospital Zhejiang University School of Medicine Hangzhou 310009 China; ^2^ Zhejiang University‐University of Edinburgh Institute (ZJU‐UoE Institute) Zhejiang University School of Medicine Zhejiang University Haining 314400 China; ^3^ Institute of Translational Medicine Zhejiang University Hangzhou 310029 China; ^4^ Department of Orthopedics The Affiliated Changzhou No.2 People's Hospital of Nanjing Medical University Changzhou 213003 China; ^5^ The Affiliated Hospital of Stomatology School of Stomatology Zhejiang University School of Medicine and Key Laboratory of Oral Biomedical Research of Zhejiang Province Hangzhou 310006 China; ^6^ Department of Radiation Oncology The Second Affiliated Hospital Zhejiang University School of Medicine Hangzhou 310009 China; ^7^ Research Center for Life Science and Human Health Binjiang Institute of Zhejiang University Hangzhou 310053 China; ^8^ The National Key Laboratory of Biobased Transportation Fuel Technology Zhejiang University Hangzhou 310027 China

**Keywords:** buthionine sulfoximine, cuproptosis‐like death, Methicillin‐resistant *Staphylococcus aureus*, pneumonia, *pseudomonas aeruginosa*, quorum sensing

## Abstract

The emergence of antibiotic‐resistant bacteria has exacerbated the challenge of treating infectious diseases. Quorum sensing (QS), a bacterial communication system regulating virulence and biofilm formation, presents a target for novel therapies. Cuproptosis death is a innovation mode of death, however, this effect may be partially inhibited by glutathione (GSH). Buthionine sulfoximine (BSO) is responsible for GSH biosynthesis and has been identified as a potential promoter of cuproptosis death. Here, Cu_2_O‐BSO NPs with lung adhesion and mucus penetration ability are synthesized by incorporating BSO onto Cu_2_O, and modifying it with DOPA and PEG. Cu_2_O‐BSO NPs demonstrated a broad‐spectrum antibacterial activity against both Gram‐positive and Gram‐negative bacteria, making it a viable treatment option for MRSA‐induced acute pneumonia. Specifically, Cu_2_O‐BSO NPs can synergistically enhance bacterial cuproptosis‐like death, hinder the QS system, eradicate biofilms, reduce the virulence of strains, stimulate the chemotaxis and phagocytosis of macrophages, and ultimately improve in mice with severe pneumonia. This research demonstrated the potential of Cu_2_O‐BSO NPs for a wide‐ranging antibacterial alternative, providing promise for addressing microbial resistance and combatting biofilm formation. Additionally, it established a target and theoretical foundation for the clinical treatment of numerous challenging cases of acute drug‐resistant bacteria.

## Graphical abstract

Copper‐based nanoparticles doped with BSO, and modified with PEG, not only can it increase the retention ability and mucosal penetration ability in the lungs, but also induce cuproptosis‐like death in bacteria and inhibit bacterial TCA cycle. It can also inhibit bacterial quorum sensing, clear biofilms, reduce bacterial virulence, and thus exert excellent therapeutic effects in acute MRSA pneumonia.

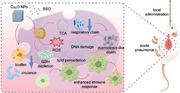

## 1 Introduction

Due to antimicrobial resistance and high virulence, infections caused by Methicillin‐resistant *Staphylococcus aureus* (MRSA) and *pseudomonas aeruginosa* (PAO1) are more complex and have a higher mortality rate.^[^ ^1^ ^]^ MRSA produces a number of virulence factors, including α‐hemolysin (Hla), phenol‐soluble modulins (PSMs), exfoliating toxins, and leukocidins, which are primarily critical for the spread and survival of the bacteria in the host.^[^ ^2^ ^]^ PAO1 demonstrates the ability to acclimate to hostile host environments through the secretion of a diverse array of virulence factors, including lipopolysaccharides, outer membrane proteins, flagella, pili, adhesins, and a type 6 secretion system.^[^ ^3^ ^]^ Moreover, the biofilms are distinguished by a protective community surrounded by a self‐generated extracellular polymer.^[^ ^4^ ^]^ The compact physical structure and the polysaccharides and nucleic acids contained in the extracellular polymeric substance (EPS) function as a defensive shield, inhibiting the infiltration of antibiotics and resulting in a notable elevation in antibiotic resistance among bacteria inhabiting the biofilm matrix.^[^ ^5^ ^]^ These virulence factors and biofilm regulation mechanisms are contingent upon cell density.^[^ ^6^ ^]^ By releasing quorum sensing (QS) autoinducers, they can help bacteria adapt to harsh host environments and thrive, leading to persistent infections in patients. Therefore, the constant discovery of novel methods and therapeutic targets for inhibiting QS systems and other virulence factors of drug‐resistant bacteria and removing biofilms is imperative.

Copper, a vital micronutrient for human health, exhibits dose‐dependent antimicrobial properties and cytotoxicity, making it a commonly utilized agent against a variety of microorganisms including gram‐negative bacteria, gram‐positive bacteria, and fungi.^[^ ^7^ ^]^ Recent studies have demonstrated the potential of copper‐based nanomaterials in chemodynamic therapy through valence transitions between Cu (I) and Cu (II) to exhibit antibacterial and anti‐biofilm properties.^[^ ^8^ ^]^ Moreover, the abnormal accumulation of copper ions in cells interferes with lipid‐acylated components of the tricarboxylic acid cycle (TCA), and ultimately inducing proteotoxic stress and cell death.^[^ ^9^ ^]^ This newly discovered mode of cell death, known as cuproptosis, allows research into cancer treatments to reach a new milestone.^[^ ^10^ ^]^ Specifically, when copper ions accumulate excessively within cells, they have the potential to interact with thioamino acids, such as cysteine, leading to the formation of Cu‐S complexes. This interaction can result in the modification of thioamino acid residues in proteins, inducing abnormal protein aggregation, thereby impairing their normal biological functions. The accumulation of copper ions disrupts the function of Fe‐S cluster proteins within mitochondria, resulting in impaired cellular energy metabolism and increased oxidative stress. Consequently, the abnormal aggregation of proteins and heightened oxidative stress contribute to cellular dysfunction and ultimately to the occurrence of cuproptosis in cells.

Researchers have also investigated the cuproptosis death of prokaryotic cells, utilizing copper‐based materials to induce a cuproptosis‐like death in bacteria for antibacterial and anti‐biofilm purposes.^[^ ^11^ ^]^ Previous research on bacterial cuproptosis‐like death has primarily focused on superficial areas like the skin and bone, neglecting deeper tissues such as the lungs. Additionally, the heavy metal toxicity caused by copper ions has evident toxic effects on liver and kidneys, which limits their use in systemic administration.^[^ ^12^ ^]^ Therefore, our study aimed to investigate the potential of copper‐based materials in the treatment of acute bacterial pneumonia through airway administration.

Bacterial pneumonia is viewed as a significant issue for public health due to its substantial mortality and morbidity.^[^ ^1^, ^13^ ^]^ MRSA and PAO1 are major contributors to acute bacterial pneumonia infection and pose significant threats to human health.^[^ ^2b^ ^]^ Pulmonary inhalation is a non‐invasive method of drug delivery through the throat and bronchus.^[^ ^14^ ^]^ Inhalation therapy enables good control of drug administration in the lungs and minimizes systemic side effects, which has great potential for treating a variety of pulmonary conditions, including tuberculosis, asthma, chronic obstructive pulmonary disease, and pulmonary infection.^[^ ^15^ ^]^ However, in the context of pulmonary administration, it is necessary for drugs to traverse the mucus layer that envelops the lung mucosa to achieve absorption. The adhesive properties of marine mussels, which are primarily attributed to the presence of Dopamine hydrochloride (DOPA) in their byssus, have been recognized as a crucial factor in facilitating wet adhesion.^[^ ^16^ ^]^ More importantly, the surface modification of nanoparticles with polyethylene glycol (PEG) has emerged as a widely adopted approach for augmenting mucus permeability.^[^ ^17^ ^]^ In conclusion, the utilization of DOPA‐inspired tissue adhesives incorporating NPs as a drug carrier, combined with PEG to modify the surface charge, presents a promising strategy to address the limitations associated with pulmonary inhalation pathways by enhancing both adhesion and penetration.

Cuprous oxide nanoparticles (Cu_2_O)‐based nanoparticles can effectively catalyze H_2_O_2_ in inflammatory sites, and produce hydrogen groups with strong antibacterial properties through Fenton like reaction, causing bacterial cell membranes to be damaged.^[^ ^18^ ^]^ Meanwhile, these nanoparticles have been demonstrated to promote the aggregation of lipid‐acylated proteins and the degradation of Fe–S proteins through elevating copper concentrations, eventually resulting in cuproptosis. Besides, these nanoparticles have also been documented to exhibit anti‐biofilm characteristics by generating reactive oxygen species (ROS), and engaging in electrostatic interactions with bacterial membranes. The presence of glutathione (GSH) in bacteria serves as a protective mechanism against oxidative stresses, thereby reducing the likelihood of cuproptosis induced by Cu_2_O. Moreover, GSH depletion can lead to increased oxidative stress in cells, change the stability or permeability of cell membrane, affect the synthesis, secretion and perception of signal molecules, and thus inhibit QS.^[^ ^19^ ^]^ Buthionine sulfoximine (BSO), an inhibitor of γ‐Glutamylcysteine synthetase responsible for GSH biosynthesis, has been identified as a potential enhancer of cuproptosis by effectively depleting intracellular GSH levels.^[^ ^9^, ^20^ ^]^ The combined action of Cu_2_O and BSO is anticipated to synergistically induce cuproptosis‐like death in bacteria.

**Scheme 1:**
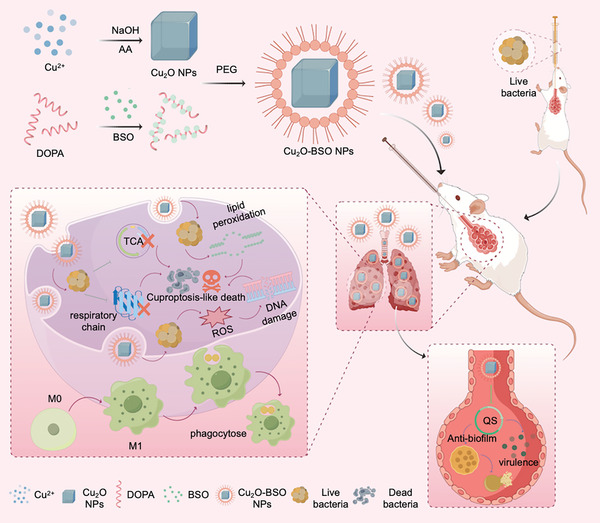
Schematic diagram of the antibacterial activity and therapeutic effect of Cu_2_O‐BSO NPs in mediating acute pneumonia. Synthetic Cu_2_O NPs and DOPA‐coated BSO were encapsulated in PEG to synthesize antibacterial nanoparticles (Cu_2_O‐BSO NPs). After intratracheal instillation, Cu_2_O‐BSO NPs can stay in the lungs, releasing Cu and BSO, which jointly induce bacterial cuproptosis‐like death, inhibit the QS system, activate the phagocytic function of macrophages, and ultimately play an antibacterial and therapeutic role in acute bacterial pneumonia.

Here, we investigate the application of intelligent Cu_2_O‐doped BSO nanoparticles (Cu_2_O‐BSO NPs) for the treatment of acute bacterial pneumonia (**Scheme** **1**). Upon inhalation, these nanoparticles exhibit efficient deposition in the lungs, thereby prolonging their duration at the infection site. The release of Cu_2_O and BSO from Cu_2_O‐BSO NPs could synergistically induce cuproptosis‐like in bacteria, thus exerting a synergistic antibacterial effect. Furthermore, they also exhibit the ability to attenuate bacterial virulence and interfere with biofilm formation by inhibitin the QS system. Collectively, our study established the concept that broad‐spectrum anti‐bacterial nanoplatforms offer a potent, safe, clinically relevant strategy for treating acute bacterial pneumonia.

## 2 Results and Discussion

### 2.1 Synthesis and Characteristics of Cu_2_O‐BSO NPs

**Figure 1:**
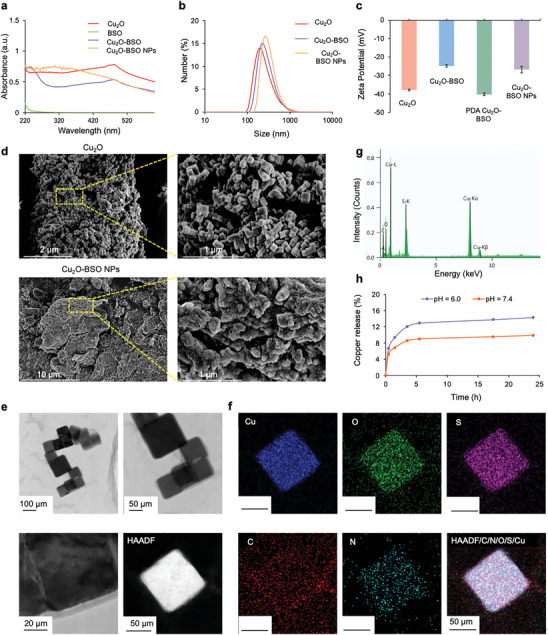
Characterization of Cu_2_O, BSO, Cu_2_O‐BSO, Cu_2_O‐BSO NPs. a) UV–vis–NIR spectra of Cu_2_O, BSO, Cu_2_O‐BSO, Cu_2_O‐BSO NPs. b) Particle sizes of Cu_2_O, Cu_2_O‐BSO, Cu_2_O‐BSO NPs. c) Zeta potentials of Cu_2_O, Cu_2_O‐BSO, PDA Cu_2_O‐BSO, Cu_2_O‐BSO NPs. d) SEM images of Cu_2_O and Cu_2_O‐BSO NPs with different magnifications. e) TEM images of Cu_2_O‐BSO NPs with different magnifications. f,g) Energy spectrum analysis of Cu_2_O‐BSO NPs (Cu, O, S, C, N). h) Release curves of copper under different conditions (pH 6.0 and 7.4).

Cu_2_O were prepared by the reduction of ascorbic acid (AA) by Copper (II) nitrate trihydrate (Cu(NO_3_)_2_·3H_2_O) in alkaline environment, then DOPA and BSO were added to modify drugs and polydopamine (PDA) on the surface of the nanoparticles, which could improve the stability of Cu_2_O NPs, and finally PEG was modified on the surface of the particles to further enhance the mucous layer penetration of Cu_2_O‐BSO NPs. The absorption curves and dynamic light scattering (DLS) of Cu_2_O, Cu_2_O‐BSO and Cu_2_O‐BSO NPs were shown in the **Figures** **1**a,b and S1 (Supporting Information). After loading the BSO, the absorption curve of Cu_2_O‐BSO NPs changed, while the hydrodynamic diameter increased, indicating the successful loading of the BSO, PDA, and PEG. Then, we measured the surface potential of particles. Figure 1c showed that the surface potential of Cu_2_O‐BSO NPs decreased after the adsorption of PEG, which was more conducive to penetrating the mucus barrier of the lungs.

The morphology of Cu_2_O and Cu_2_O‐BSO NPs was next observed by scanning electron microscopy (SEM). Figure 1d depicted that Cu_2_O nanoparticles were regular cubes with a size of ≈100 nm, and their surface were covered by a polymer layer after the modification. The transmission electron microscopy (TEM) was further used to determine the morphology and element distribution of Cu_2_O‐BSO NPs. In Figure 1e, the nanoparticles were basically cube‐like, consistent with SEM. The results of energy spectrum analysis indicated that the NPs contained Cu, O, S, C, N elements, and were basically evenly distributed on the cubes, which further confirmed the successful preparation of Cu_2_O and the loading of the BSO (Figure 1f,g). Additionally, we measured the release curves of copper under different conditions (Figure 1h). It suggested that copper ions could be slowly released into the environment after modification, and the release rate was further improved under the weakly acidic environment of inflammation.

### 2.2 Pulmonary Retention and Mucosal Penetration of Cu_2_O‐BSO NPs

**Figure 2:**
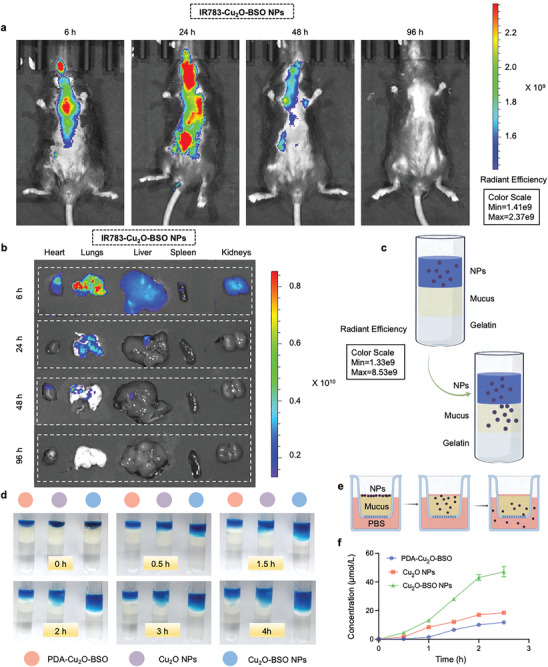
Pulmonary retention and mucosal penetration of Cu_2_O‐BSO NPs. a) Representative in vivo fluorescence images of mice at 6, 24, 48, and 96 h after airway injection of 50 µL IR783‐Cu_2_O‐BSO NPs. b) Representative ex vivo fluorescence images of heart, lungs, liver, spleen, and kidneys at specified time after airway injection of IR783‐Cu_2_O‐BSO NPs. c,d) Model diagram and visual observation of the penetration of PDA‐Cu_2_O‐BSO, Cu_2_O NPs, and Cu_2_O‐BSO NPs in the artificial mucus layer at 0, 0.5, 1.5, 2, 3, and 4 h. e,f) Model map and quantitative analysis of transwell assay for detecting the content of copper ions permeating artificial mucosa.

Considering the excellent tissue adhesion of DOPA, the IVIS imaging system was applied to assess the retention time of Cu_2_O‐BSO NPs in the lungs. IR783 is a dye commonly used in the biomedical field, which has good near‐infrared absorption properties and is widely used in vivo imaging.^[^ ^21^ ^]^ Accordingly, the IR783 dye was coupled to Cu_2_O‐BSO NPs (IR783‐Cu_2_O‐BSO NPs), and it was intratracheally instilled into the lungs of mice. Fluorescence imaging was performed at 6, 24, 48, and 96 h to analyze the fluorescence intensity in the lungs at various time points both in vivo and ex vivo. First, strong fluorescence intensity of IR783‐Cu_2_O‐BSO NPs was observed in the lungs at 6, 24, and 48 h, demonstrating that Cu_2_O‐BSO NPs can remain in the lungs for more than 48 h (**Figure** **2**a). When fluorescence intensity was measured ex vivo, it was highly similar to that measured in vivo, implying that fluorescence was mainly concentrated in the lungs at 6, 24, and 48 h (Figure 2b). These results collectively indicated that Cu_2_O‐BSO NPs exhibit excellent lung adhesion.

As shown in Figure 2c, we used an artificial mucus model to investigate the effect of PEG on the permeability of Coomassie Brilliant Blue‐labeled Cu_2_O‐BSO NPs through mucous membranes.^[^ ^22^ ^]^ In short, equal amounts of Cu_2_O NPs, PDA‐Cu_2_O‐BSO (PEG unmodified) or Cu_2_O‐BSO NPs were added to artificial mucus respectively, and the penetration in artificial mucus was recorded at 0, 0.5, 1.5, 2, 3, and 4 h. Figure 2d suggested that the Cu_2_O‐BSO NPs group has a faster penetration rate than the PDA‐Cu_2_O‐BSO and Cu_2_O NPs groups, indicating that PEG significantly enhanced the mucosal permeable ability. More importantly, we further used transwell to detect the content of copper ions that have penetrated to quantify the mucosal permeable ability (Figure 2e). Similar to previous results, the Cu_2_O‐BSO NPs group had more copper ions that have penetrated, suggesting a strongest mucosal permeable ability (Figure 2f).

In a nutshell, the above results all proved that Cu_2_O‐BSO NPs modified by DOPA and PEG have excellent lung retention and mucosal penetration capabilities.

### 2.3 Anti‐MRSA Activity of Cu_2_O‐BSO NPs In Vitro

**Figure 3:**
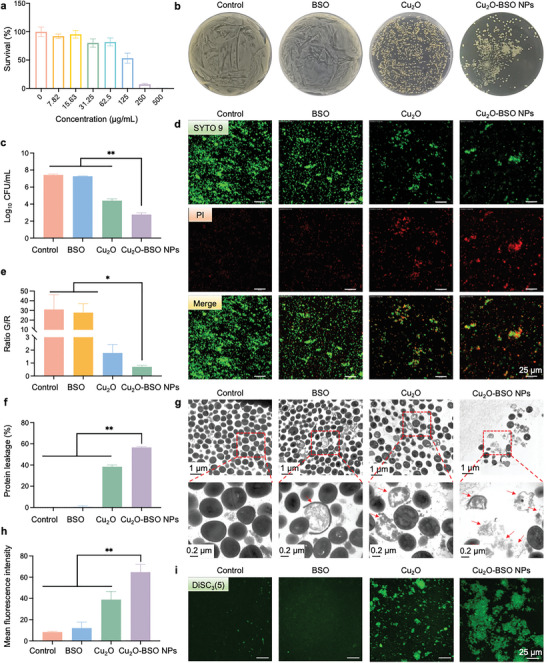
Antibacterial potential to planktonic MRSA in vitro. a) Survival rate of MRSA after treatment of Cu_2_O‐BSO NPs. b,c) Agar plate photos and quantitative analysis of MRSA after treatment of blank TSB, BSO, Cu_2_O and Cu_2_O‐BSO NPs, respectively. d,e) Representative fluorescence photos and quantitative analysis of MRSA stained with SYTO 9 (green, live bacteria) and PI (red, dead bacteria) after different treatments. f) Protein leakage for MRSA with corresponding treatments. g) Representative TEM images (top) and corresponding higher magnification images (bottom) of MRSA after different treatment. Red arrows are damaged bacteria (bacterial wall ruptures and bacterial contents leak). h,i) Quantitative analysis and representative fluorescence photos of bacterial membrane potential detected by DiSC_3_(5) dyes. The stronger green fluorescence, the higher depolarization of the bacterial membrane.

Due to its resistance to multiple antibiotic classes, MRSA is a pathogen associated with severe morbidity and mortality.^[^ ^23^ ^]^ The anti‐MRSA effect of Cu_2_O‐BSO NPs and ampicillin was first determined by minimum inhibitory concentration (MIC). A 250 µg mL^−1^ MIC was found for Cu_2_O‐BSO NPs against MRSA while the MIC of ampicillin was >250 µg mL^−1^ (**Figure** **3**a; Figure S2, Supporting Information). Subsequently, we further compared the antibacterial activities of the Cu_2_O, BSO, and Cu_2_O‐BSO NPs groups (Figure 3b,c), and these results indicated that BSO exhibited virtually no significant antibacterial activity, while Cu_2_O demonstrated a comparatively superior antibacterial effect, and Cu_2_O‐BSO NPs exhibited the most optimal antibacterial activity. Afterward, we further conducted experiments on the bacterial live/dead staining. Consistent with the aforementioned results, both Cu_2_O and Cu_2_O‐BSO NPs exhibited obvious antibacterial effects, with the strongest red fluorescence observed in the Cu_2_O‐BSO NPs group, indicating the best antibacterial efficacy (Figure 3d,e). These results concluded that the isolated BSO alone exhibited no significant antibacterial activity, however, it can exert a synergistic effect with Cu_2_O to achieve an efficient antimicrobial action.

Considering the variable release rates of Copper at different pH levels, we investigated the ROS promoting capability of Cu_2_O‐BSO NPs at pH 6 and 7.4, alongside the viability status of MRSA. The findings indicated that at pH 6, the Cu_2_O‐BSO NPs group generated a higher amount of ROS, resulting in increased bacterial mortality, as evidenced by more intense red fluorescence. This phenomenon may be attributed to the enhanced release of Copper (Figures S3 and S4, Supporting Information).

As bacteria are separated from the environment by membranes, making their integrity critically vital for bacterial viability, virulence, and function.^[^ ^24^ ^]^ Intriguingly, the Bradford assay revealed that MRSA treated with Cu_2_O or Cu_2_O‐BSO NPs exhibited evident protein leakage (Figure 3f). To further investigate whether the potential antibacterial mechanism was related to the disruption of bacterial membrane integrity, the morphology of MRSA was examined using TEM. Figure 3g depicted that most of the bacteria treated with blank Tryptic Soy Broth (TSB) and BSO maintained their original morphology, such as intact cell walls, smooth cell membranes, and uniform dense cytoplasm. In contrast, treatment with Cu_2_O and Cu_2_O‐BSO NPs led to bacterial lysis, cell wall fragmentation, cell membrane shrinkage, and cellular contents leakage. Membrane potential refers to the voltage difference formed on the membrane surface, which can be used to understand the integrity of bacterial membranes.^[^ ^25^ ^]^ The 3,3′‐dipropylthiadicarbocyanine iodide (DiSC_3_(5)) fluorescent probe is commonly used to measure membrane potential.^[^ ^26^ ^]^ When the bacterial membrane loses its integrity, the resulting membrane disorder will manifest as a depolarization of the membrane potential, contributing to an increase in green fluorescence intensity. The detection results showed a strong green fluorescence in the Cu_2_O group, suggesting a severe bacterial membrane disruption while the Cu_2_O‐BSO NPs group exhibited a stronger green fluorescence, demonstrating a more pronounced bacterial membrane disruption (Figure 3h,i). Subsequently, N‐phenyl‐1‐naphthylamine (NPN) method was used to determine the permeability of the bacterial outer membrane, with stronger blue fluorescence indicating higher permeability. The results showed that Cu_2_O‐BSO NPs group had the strongest blue fluorescence, indicating the most serious leakage (Figure S5, Supporting Information). In conclusion, Cu_2_O‐BSO NPs were expected to exert an antibacterial effect by disrupting the integrity of bacterial membranes. Prior studies have confirmed that ROS can interact with various bacterial components, including proteins, lipids, and polysaccharides, causing oxidative stress on bacterial membranes, disruption of plasma membrane integrity, and eventual cell death.^[^ ^27^ ^]^ Cu_2_O is thought to impact the respiratory chain, resulting in elevated ROS levels.^[^ ^10^ ^]^ Additionally, BSO serves as a potent inhibitor of glutathione cysteine synthase biosynthesis, reducing the content of GSH and disturbing the homeostasis of intracellular ROS, thus achieving enhanced oxidative stress‐induced cell death.^[^ ^28^ ^]^ Therefore, the bacterial membrane disruption may be caused by a large amount of ROS generated by the combined action of Cu_2_O and BSO.

### 2.4 Anti‐biofilm Properties of Cu_2_O‐BSO NPs In Vitro

**Figure 4:**
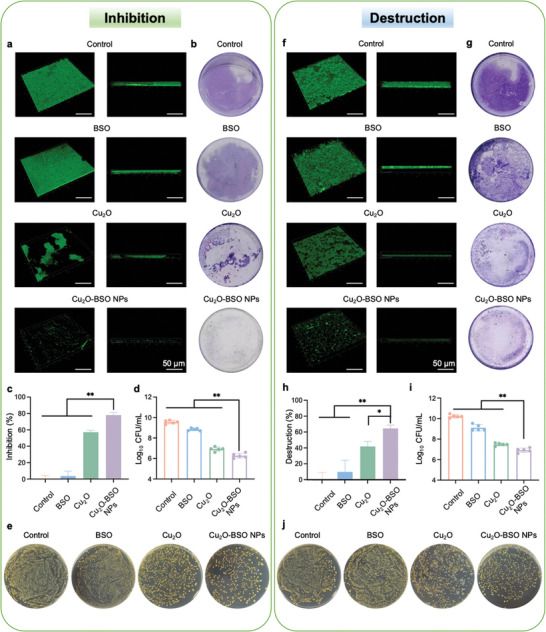
Anti‐MRSA biofilm capability in vitro. a) 3D structure of MRSA immature biofilms (stained with SYTO 9) after treatment of blank TSB, BSO, Cu_2_O and Cu_2_O‐BSO NPs, respectively at 0 h by CLSM (left, top view; right, side view). b,c) Photographs of crystal violet dyeing and inhibition rate of immature biofilm after different treatment at 0 h. d,e) Count of viable bacteria and agar plate photographs of MRSA immature biofilms in different groups. f) 3D structure of MRSA mature biofilms (stained with SYTO 9) after different treatments at 24 h by CLSM (left, top view; right, side view). g,h) Photographs of crystal violet dyeing and destruction rate of mature biofilm after corresponding treatments. i,j) Count of viable bacteria and agar plate photographs of MRSA mature biofilms in different groups.

Microbial biofilms contain 3D structures, extracellular matrix, and interbacterial signals that are responsible for promoting bacterial community growth, persistence, and antibiotic resistance.^[^ ^29^ ^]^ First, we verified the anti‐biofilm ability of different concentrations of Cu_2_O‐BSO NPs, and the results showed that Cu_2_O‐BSO NPs had excellent ability to inhibit biofilm formation and destroy mature biofilm at 500 µg mL^−1^ (Figure S6, Supporting Information). Subsequently, the anti‐biofilm activity was evaluated in the presence of blank TSB, BSO, Cu_2_O or Cu_2_O‐BSO NPs, respectively. Confocal laser scanning microscopy (CLSM) was conducted to detect the 3D structure of biofilms. As anticipated, after corresponding treatment at the early time (0 h), the biofilms were severely destroyed after Cu_2_O‐BSO NPs treatment, displaying sporadic and thin characteristics (**Figure** **4**a). Similarly, crystal violet staining further demonstrated that the biofilms maintained their structural integrity following blank TSB and BSO treatment, suggesting that the dense biofilm structure was resistant to disruption by BSO. Conversely, biofilms treated with Cu_2_O and Cu_2_O‐BSO NPs were notably thinner and had less biofilm biomass, particularly the latter (Figure 4b,c). Furthermore, the quantitative analysis of viable bacteria in the biofilms was performed using the spread plate method (SPM). In short, the SPM method refers to the method of coating a bacterial suspension on a TSB agar medium to obtain the number of bacterial clones. The results of bacterial colony forming units (CFU) suggested that both Cu_2_O and Cu_2_O‐BSO NPs effectively killed a majority of the bacteria in the biofilms, while the CFU of MRSA treated with BSO did not decrease obviously (Figure 4d,e). These findings all demonstrated that Cu_2_O‐BSO NPs can effectively inhibit the formation of MRSA biofilms. To further gain a comprehensive understanding of its superior anti‐biofilm properties, CLSM (Figure 4f), crystal violet staining (Figure 4g,h), and SPM (Figure 4i,j) were re‐performed to assess the removal capability of Cu_2_O‐BSO NPs on mature biofilms. Intriguingly, after post‐treatment (24 h) of the biofilms, the aforementioned results were almost consistent with the biofilm inhibitory experiments, demonstrating that Cu_2_O and Cu_2_O‐BSO NPs exhibited outstanding abilities to disrupt mature biofilms, with the latter being superior. To further explore the specific effect of Cu_2_O‐BSO NPs on EPS, Calcofluor white dye was used. Figure S7 (Supporting Information) depicted that EPS in Cu_2_O‐BSO NPs group was least, indicating the best anti‐EPS activity. In a nutshell, Cu_2_O‐BSO NPs not only can inhibit the formation of immature biofilms but also can disrupt mature biofilms.

ROS can disrupt the EPS of infectious biofilms, taking part in removing biofilms.^[^ ^30^ ^]^ Moreover, ROS can also cause significant oxidative stress in bacteria, resulting in protein denaturation and enzyme deactivation. In addition, ROS can directly inhibit the synthesis and efflux of autoinducible peptide (AIP) through DNA damage, exerting anti‐QS effects and further inhibiting the formation of bacterial biofilms.^[^ ^31^ ^]^ Concurrently, BSO can degrade GSH in biofilms, weakening the resistance of biofilms to antimicrobial drugs. The aforementioned evidence may confirm that Cu_2_O and BSO have synergistic anti‐biofilm effects.

### 2.5 Regulation of MRSA Transcriptome by Cu_2_O‐BSO NPs

**Figure 5:**
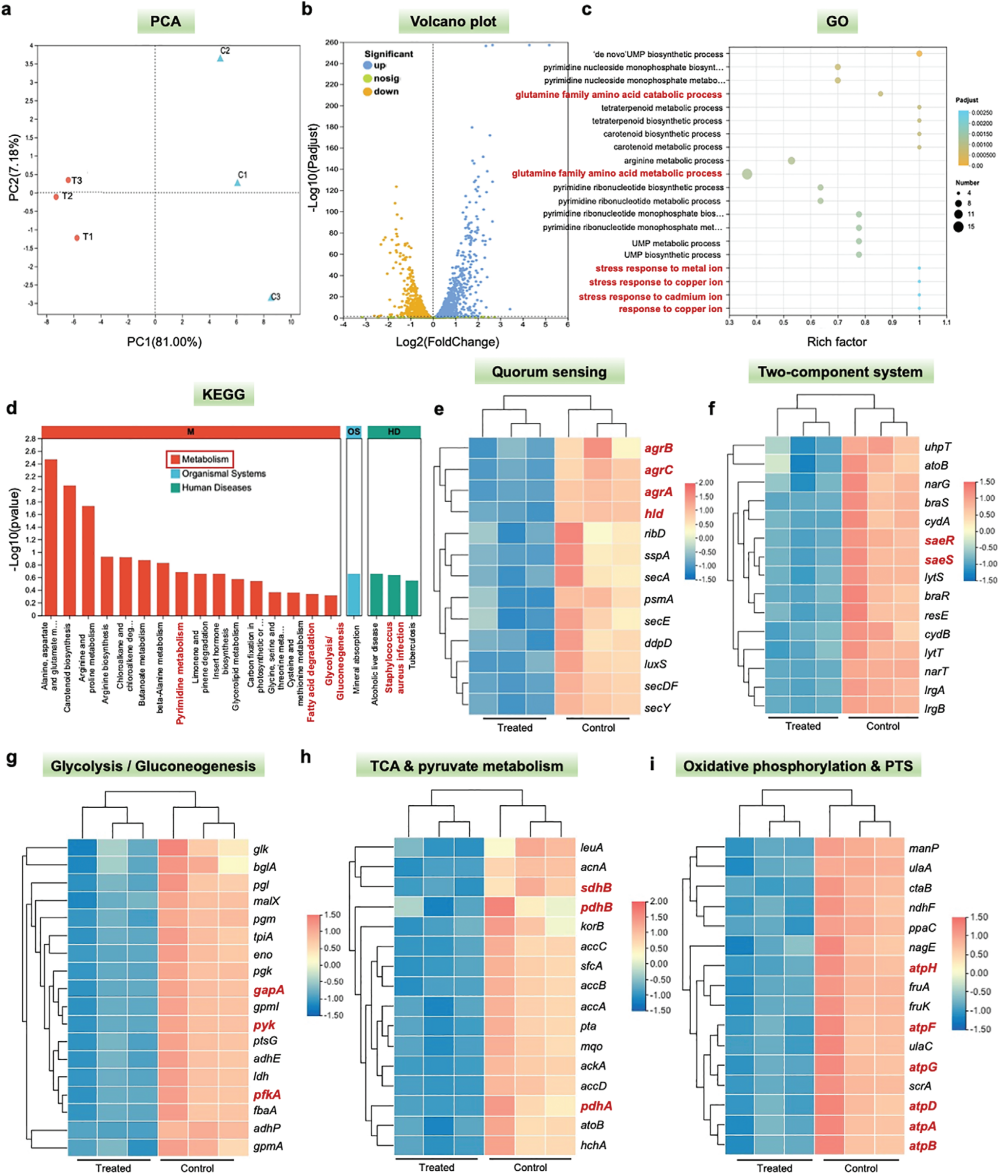
Regulation of the MRSA transcriptome by Cu_2_O‐BSO NPs. a) PCA analysis of blank TSB (C1, C2, C3) group and Cu_2_O‐BSO NPs (T1, T2, T3) group. b) Volcano plot analyses of total DEGs in MRSA after treatment with blank TSB or Cu_2_O‐BSO NPs. Orange and blue dots represent downregulated and upregulated genes, respectively. Green dots represent the genes that were not statistically different. c) GO enrichment analysis of DEGs. d) KEGG enrichment analysis of DEGs. e) Heat map of genes associated with QS. f) Heat map of genes associated with TCS. g) Heat map of genes associated with glycolysis/gluconeogenesis. h) Heat map of genes associated with TCA cycle and pyruvate metabolism. i) Heat map of genes associated with oxidative phosphorylation and PTS.

Transcriptomic sequencing (RNA‐seq) analysis of MRSA was conducted to investigate potential targets and mechanisms of antibacterial action. Initially, principal component analysis (PCA) analysis confirmed the reliability of the sequencing results (**Figure** **5**a). Moreover, after treatment with Cu_2_O‐BSO NPs, Figures 5b and S8 (Supporting Information) depicted that a total of 1459 differentially expressed genes (DEGs) were identified in the MRSA gene expression profile, with a statistically significant difference observed in the volcano plot analysis (log2(treated/control) > 1 or log2(treated/control) < ‐1). Among these, 747 genes were up‐regulated and 712 genes were down‐regulated. Gene Ontology (GO) enrichment analysis and chord diagram revealed that Cu_2_O‐BSO NPs could affect processes such as glutamate metabolism and catabolism, and stress to copper ions (Figure 5c; Figure S9, Supporting Information). Kyoto Encyclopedia of Genes and Genomes (KEGG) enrichment analysis and chord diagram indicated that Cu_2_O‐BSO NPs primarily affect metabolism, including glutamate metabolism, pyrimidine metabolism, fatty acid degradation, and glycolysis (Figure 5d; Figure S10, Supporting Information). The heat maps were further utilized to analyze the consequence of Cu_2_O‐BSO NPs on specific bacterial functions.

The QS mechanism of bacteria allows them to coordinate behavior at the population level.^[^ ^32^ ^]^ The process of QS is a bacterial cell‐to‐cell communication that controls collective behavior, for instance, biofilm formation, virulence factor production, drug resistance establishment, and bacterial movement.^[^ ^33^ ^]^ Thus, QS has been targeted to control bacterial infections. The two‐component system (TCS) is a primary signaling mechanism by which bacteria sense environmental changes and respond to adapt and survive.^[^ ^34^ ^]^ *Agr* system (*agrA/B/C/D*) is the core part of MRSA's QS system, which controls the pathogenicity of bacteria by regulating the expression of virulence factors and the formation of bacterial biofilm.^[^ ^35^ ^]^ The *hld* gene is one of the genes involved in α‐hemolysin synthesis in MRSA. α‐hemolysin is an important virulence factor that can damage the membranes of host cells, especially red blood cells, white blood cells, and endothelial cells, thereby helping bacteria invade host tissues and evade the host immune response.^[^ ^36^ ^]^ *Sae* system is a typical TCS consisting of *saeR* (receptor kinase) and *saeS* (response regulator). Specifically, *saeR/S* is closely related to the QS system (*agr*), synergistically regulate bacterial virulence factor expression, cell wall synthesis, and immune escape mechanisms.^[^ ^37^ ^]^ The heat maps analysis revealed down‐regulation of genes associated with QS, TCS, and *Staphylococcus aureus* infection (such as *agrA, agrB, agrC, hld, saeR, and saeS*) following treatment with Cu_2_O‐BSO NPs. This also suggested that Cu_2_O‐BSO NPs may block QS and TCS, weaken environmental adaptability, diminish bacterial virulence and infectivity, and enhance susceptibility to antimicrobial agents (Figure 5e,f; Figures S11 and S12, Supporting Information). Additionally, examination of MRSA's hemolytic activity post‐treatment with Cu_2_O‐BSO NPs demonstrated an evident decrease, providing further evidence of the successful attenuation of virulence (Figure S13, Supporting Information).

Cuproptosis is a novel cell‐death phenomenon triggered by the excessive influx of copper ions into cells, resulting in their reduction of copper ions to the more state of toxic Cu(I).^[^ ^9^ ^]^ An excess of intracellular copper can also impede the TCA cycle, induce the accumulation of lipid peroxides and promote the death of MRSA.^[^ ^11^ ^]^ The TCA cycle is one of the core processes of bacterial energy metabolism, responsible for the oxidation of pyruvate and the generation of ATP, NADH and FADH₂, providing the energy needs of bacteria.^[^ ^38^ ^]^ Succinate dehydrogenase (*sdhA/B*) is involved in the regulation of the TCA cycle and is responsible for converting succinate to fumaric acid, which plays an important role in the bacterial respiratory chain. The pyruvate dehydrogenase complex (*pdhA/B/C/D*) is a key enzyme complex that converts pyruvate to acetyl‐CoA, a process that connects glycolysis and the TCA cycle.^[^ ^39^ ^]^ Excessive copper will also inhibit the activity of key glycolysis enzymes (*gapA, pyk, pfk*), resulting in a decrease in the rate of glycolysis and affecting the production of ATP (*atpA/B/D/G/F/H*).^[^ ^40^ ^]^ Remarkably, the heat maps in Figures 5g–i and S12 (Supporting Information) suggested that genes related to TCA cycle, pyruvate metabolism, glycolysis and oxidative phosphorylation (such as *gapA, pyk, pfkA, sdhB, pdhA, pdhB, and atpA/B/D/G/F/H*) were largely down‐regulated, resulting in a phenomenon known as bacterial cuproptosis‐like death.

In the process of lipid peroxidation induced by cuproptosis‐like death, a large quantity of ROS is generated, exerting significant oxidative stress on cells, and then contributing to protein denaturation, enzyme deactivation, and DNA damage.^[^ ^31^ ^]^ As a result, AIP synthesis and efflux are inhibited, anti‐QS activity is exerted, biofilm formation is suppressed, and bacterial virulence is reduced. Therefore, the anti‐QS effect and anti‐biofilm activity of Cu_2_O‐BSO NPs may be attributed to the ROS produced by cuproptosis‐like death. As a result of blocking the QS system, bacteria can also suffer from oxidative stress, further amplifying the effects of cuproptosis‐like death in the process. The findings indicated that Cu_2_O‐BSO NPs were capable of achieving complete sterilization through ROS‐mediated oxidative stress toxicity. This process is associated with cuproptosis‐like effects that TCA cycle and bacterial respiration, thereby suppressing QS and exerting anti‐biofilm activity. Additionally, these nanoparticles disrupt the integrity of bacterial membranes and induce DNA damage and protein leakage in bacterial cells.

### 2.6 Cuproptosis‐Like Death of MRSA In Vitro

**Figure 6:**
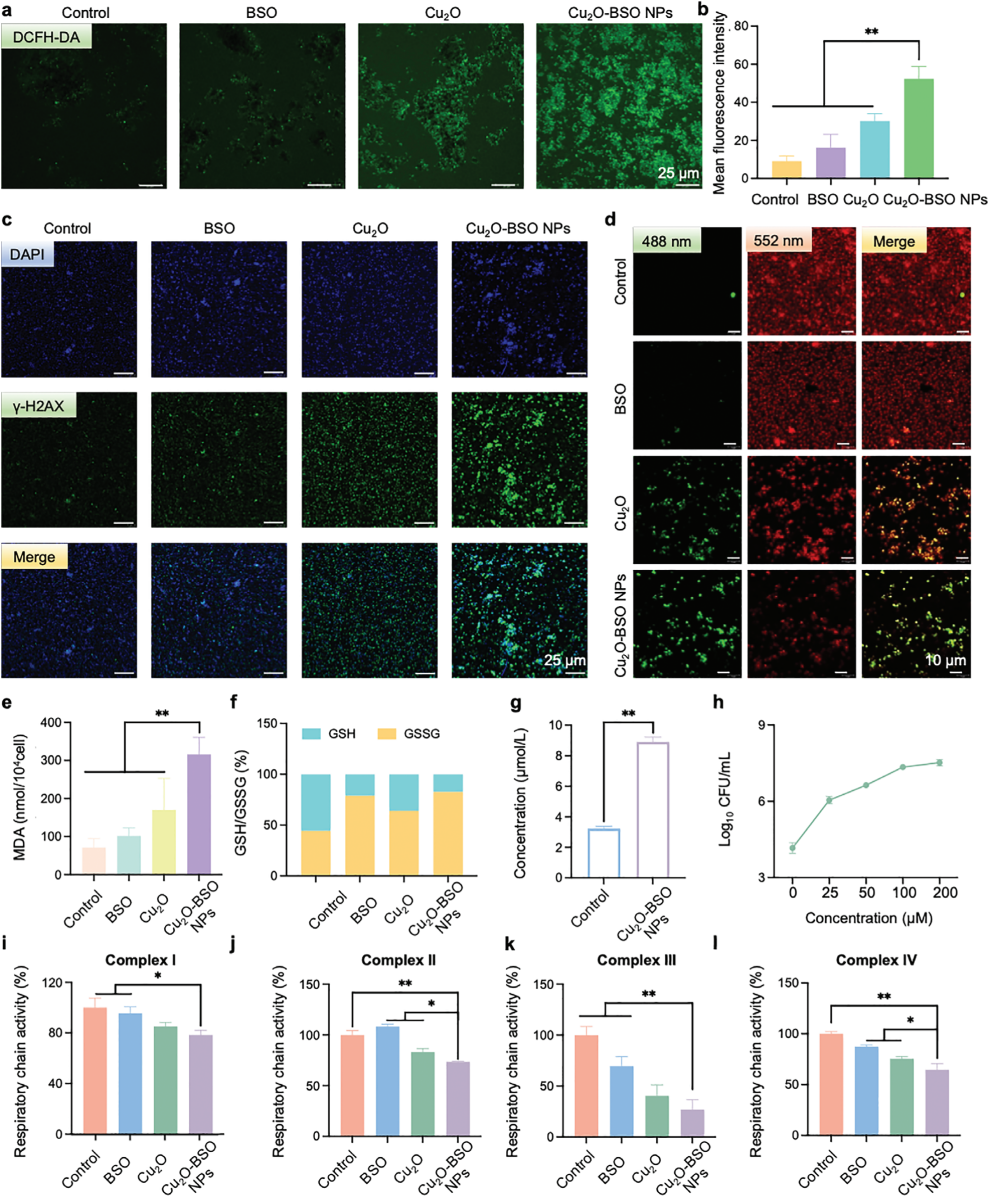
Cuproptosis‐like death of MRSA in vitro. a,b) DCFH‐DA staining fluorescence photographs and quantitative analysis of ROS (green) of MRSA after treatment with blank TSB, BSO, Cu_2_O, and Cu_2_O‐BSO NPs, respectively. c) The γ‐H2AX focus (green) in MRSA after corresponding treatments analyzed by immunofluorescence staining. d) The lipid peroxidation level of MRSA after different treatment was detected with a BODIPY581/591‐C11 probe. The fluorescence changes from red to green during lipid peroxidation. e) The lipid peroxidation level of MRSA in different groups was detected with an MDA kit. f) Ratio of GSH/GSSG after corresponding treatments. g) Copper concentration statistics of control group and Cu_2_O‐BSO NPs group. h) Effect of cuproptosis death inhibitor (EDTA) on anti‐MRSA activity of Cu_2_O‐BSO NPs.(i–l) Influence of blank TSB, BSO, Cu_2_O and Cu_2_O‐BSO NPs on the activity of bacterial respiratory chain complexes I, II, III, and IV.

The redox state of bacteria is influenced by the production of ROS and the antioxidant defense system.^[^ ^41^ ^]^ Overproduction of ROS leads to oxidative stress or even oxidative damage when consumption of antioxidant defenses is exceeded. In the aforementioned study, we speculated that the antibacterial and anti‐biofilm effects of Cu_2_O‐BSO NPs were associated with ROS. Moreover, cuproptosis‐like death can obviously affect the redox levels of MRSA. Accordingly, the DCFH‐DA probe was used to detect ROS levels in MRSA, and we observed a≈5.8‐fold increase of green fluorescence intensity in the Cu_2_O‐BSO NPs group, which was much higher than the≈3.3‐fold increase in the Cu_2_O group (**Figure** **6**a,b). This also explained the reason that the antibacterial and anti‐biofilm capabilities of Cu_2_O‐BSO NPs were superior to those of the Cu_2_O group. H2AX is rapidly phosphorylated to form γ‐H2AX after DNA double‐strand breaks, allowing the assessment of DNA damage by detecting the green fluorescence intensity of γ‐H2AX.^[^ ^42^ ^]^ Figure 6c showed that Cu_2_O‐BSO NPs exhibited the strongest green fluorescence, suggesting the most severe DNA breaks. In addition to investigating oxidative stress, our study delved into the potential for MRSA to undergo cuproptosis‐like death. A pivotal signal of cuproptosis death is the accumulation of lipid peroxides, which is a consequence of cell apoptosis.^[^ ^11b^ ^]^ Initially, the level of membrane lipid peroxidation in MRSA was primarily determined by the BODIPY581/591‐C11 probe. When polyunsaturated butadiene moiety is oxidized, BODIPY581/591‐C11 emits bright green fluorescence instead of its normal bright red fluorescence. Figure 6d showed that the control group and BSO group manifested stronger red fluorescence, while the Cu_2_O and Cu_2_O‐BSO NPs groups manifested stronger green fluorescence. Moreover, the green fluorescence was strongest in the Cu_2_O‐BSO NPs group. Malondialdehyde (MDA), a lipid peroxidation product, is widely used in biological and medical research, especially in the study of oxidative stress and cell damage. Similarly, MDA experiments indicated that Cu_2_O‐BSO NPs could enhance lipid peroxidation by≈3.4 times (Figure 6e). Considering that BSO has the function to inhibit the synthesis of GSH, the ratio of GSH/GSSG was examined, and the results also confirmed that both the BSO and Cu_2_O‐BSO NPs groups have the ability to disrupt the equilibrium of the GSH/GSSG ratio (Figure 6f). Afterward, copper ions detecting experiments demonstrated evident copper accumulation in the Cu_2_O‐BSO NPs group (Figure 6g). Ethylene diamine tetraacetic acid (EDTA), a divalent ion chelator, has been shown to form complexes with metal ions like Cu (II) to inhibit metal‐dependent enzymatic reactions. EDTA is therefore being utilized as an inhibitor of cuproptosis‐like death to ascertain its role as a potential mechanism for bacterial eradication. Conclusively, EDTA can inhibit the bactericidal activity of Cu_2_O‐BSO NPs in a concentration‐dependent manner (Figure 6h). Previous research has established that copper‐based nanoparticles have a notable impact on the downregulation of the TCA in bacteria. The findings presented in Figure 6i–l illustrated that Cu_2_O‐BSO NPs can simultaneously suppress the activity of respiratory chain complexes I, II, III, and IV, suggesting its ability to disrupt bacterial respiratory chain function. In brief, these results intimated that Cu_2_O‐BSO NPs may mediate bacterial cuproptosis‐like death, characterized by increased production of ROS, disruption of GSH homeostasis, peroxidation of lipids, and inhibition of the respiratory chain.

### 2.7 Anti‐PAO1 Effect and Mechanism of Cu_2_O‐BSO NPs

**Figure 7:**
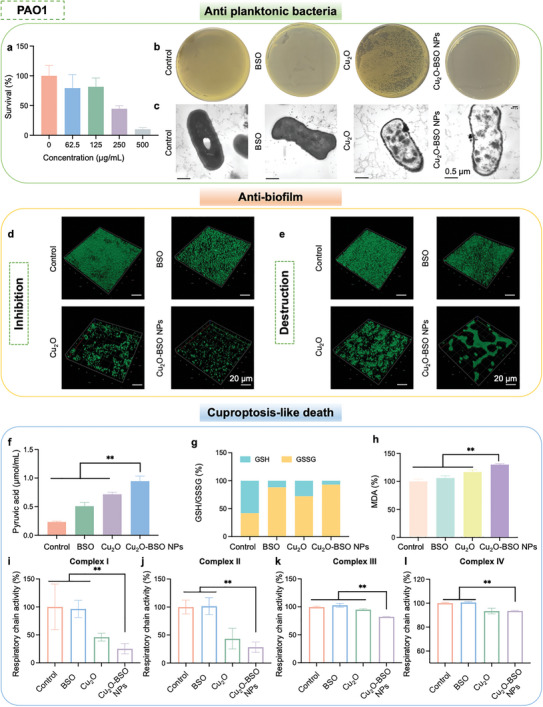
Anti‐PAO1 effect and mechanism of Cu_2_O‐BSO NPs. a) Survival rate of PAO1 after treatment of Cu_2_O‐BSO NPs. b) Agar plate photos of PAO1 after treatment of blank TSB, BSO, Cu_2_O and Cu_2_O‐BSO NPs, respectively. c) Representative TEM images of PAO1 after different treatment. d,e) 3D structure of PAO1 immature biofilms d) and mature biofilms e) after corresponding treatment at 0 and 24 h by CLSM. f–h) Statistical analysis of pyruvate content (f), ratio of GSH/GSSG (g) and detection of MDA (h) in each group. i–l) The activity of bacterial respiratory chain complexes I, II, III, and IV in different groups.

After successfully verifying the excellent antibacterial and anti‐biofilm effects of Cu_2_O‐BSO NPs on Gram‐positive bacteria (MRSA), we further investigated the effect of Cu_2_O‐BSO NPs on Gram‐negative bacteria such as PAO1. First, we confirmed that the MIC of Cu_2_O‐BSO NPs against PAO1 was 500 µg mL^−1^ while the MIC of ampicillin was >500 µg mL^−1^ (**Figure** **7**a; Figure S14, Supporting Information). Figure 7b depicted that Cu_2_O‐BSO NPs exhibited the most outstanding anti‐PAO1 effect among all groups. Subsequently, TEM was used to further explore the changes in the morphology of PAO1 (Figure 7c). TEM results showed that BSO had no significant effect on the morphology of PAO1, with only slight deformation. Cu_2_O could cause leakage of the contents of PAO1, while Cu_2_O‐BSO NPs caused the most evident cell wall disruption and leakage of intracellular contents. Excitingly, CLSM verified that Cu_2_O‐BSO NPs have both excellent abilities to inhibit biofilm formation and destroy mature biofilms (Figure 7d,e). In terms of cuproptosis‐like death, Cu_2_O‐BSO NPs can lead to obvious accumulation of pyruvate (Figure 7f), imbalance in the ratio of GSH and GSSG (Figure 7g), lipid peroxidation (Figure 7h), and apparent inhibition of respiratory chain I‐IV (Figure 7i–l). In summary, the above research results indicated that Cu_2_O‐BSO NPs have outstanding antibacterial and anti‐biofilm effects on both Gram‐positive and Gram‐negative bacteria, and can cause both to undergo cuproptosis‐like death. These experimental results also laid a theoretical foundation for the broad‐spectrum antibacterial effect of Cu_2_O‐BSO NPs.

### 2.8 Activation of Macrophage Response by Cu_2_O‐BSO NPs In Vitro

**Figure 8:**
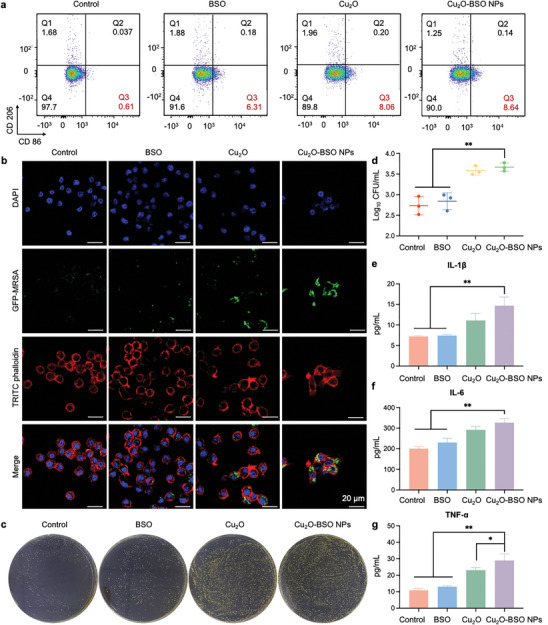
Activation of macrophage response by Cu_2_O‐BSO NPs in vitro. a) Typical scatter plots of MH‐S surface markers CD86 (M1 macrophage marker) and CD206 (M2 macrophage marker) detected by flow cytometry. b) Typical images of MH‐S engulfing a bacterium. The green fluorescence represents GFP‐MRSA, the red fluorescence represents MH‐S, and the blue fluorescence represents the nucleus. The stronger the green fluorescence, the stronger the phagocytosis of MH‐S. c,d) Representative SPM photographs and CFU count of MRSA engulfed by MH‐S. e–g) ELISA results indicating levels of cytokines (IL‐1β, IL‐6, and TNF‐α) secreted by MH‐S in different groups.

Macrophages serve as the primary immune response against intracellular pathogens by recognizing, engulfing, and killing invading bacteria, as well as up‐regulating antimicrobial mechanisms leading to bacterial lysis.^[^ ^43^ ^]^ Macrophages exhibit a high degree of plasticity, capable of dynamically adapting to changes in the microenvironment through M1/M2 polarization in response to tissue infection or inflammation.^[^ ^44^ ^]^ ROS have been confirmed to enhance macrophage polarization toward the M1 phenotype, promoting pro‐inflammatory and bactericidal effects.^[^ ^45^ ^]^ Considering that Cu_2_O‐BSO NPs can produce plenty of ROS, we further investigated the impact of Cu_2_O‐BSO NPs on macrophage function. As demonstrated in Figure S15 (Supporting Information), both Cu_2_O and Cu_2_O‐BSO NPs significantly increased the level of ROS in mouse alveolar macrophages (MH‐S), while other treatment groups did not exhibit such effects. Furthermore, we further examined whether the increased ROS altered macrophage phenotype. It is widely accepted that the activation of macrophages is commonly characterized by a set of surface and genetic markers, namely CD206 as an anti‐inflammatory M2 state and CD86 as a pro‐inflammatory M1 state.^[^ ^46^ ^]^ After various treatments, the percentage of M1 macrophages in the BSO, Cu_2_O, and Cu_2_O‐BSO NPs groups was≈6.31%, ≈8.06%, and ≈8.64%, respectively (**Figure** **8**a). These data concluded that Cu_2_O‐BSO NPs can promote macrophage polarization toward the M1 phenotype by increasing intracellular ROS levels.

To further investigate the impact of Cu_2_O‐BSO NPs on macrophage function via ROS‐mediated M1 polarization, CLSM was employed to assess macrophage phagocytosis. Macrophages treated with Cu_2_O and Cu_2_O‐BSO NPs demonstrated increased phagocytosis of MRSA (green fluorescence) compared to other groups, with Cu_2_O‐BSO NPs displaying a more pronounced effect, implying its superior phagocytosis‐enhancing properties (Figure 8b). Additionally, quantitative assessment of intracellular live bacteria by SPM further supported this conclusion (Figure 8c,d). Subsequently, enzyme‐linked immunosorbent assay (ELISA) experiments were conducted to evaluate the release of inflammatory cytokines by MH‐S after corresponding treatments (Figure 8e–g). Both Cu_2_O and Cu_2_O‐BSO NPs apparently elevated the levels of pro‐inflammatory cytokines interleukin‐1β (IL‐1β), interleukin‐6 (IL‐6), and tumor necrosis factor‐α (TNF‐α), representing those macrophages can exert a more potent bactericidal activity through pro‐inflammatory actions.

### 2.9 Antibacterial Effects of Cu_2_O‐BSO NPs in vivo

**Figure 9:**
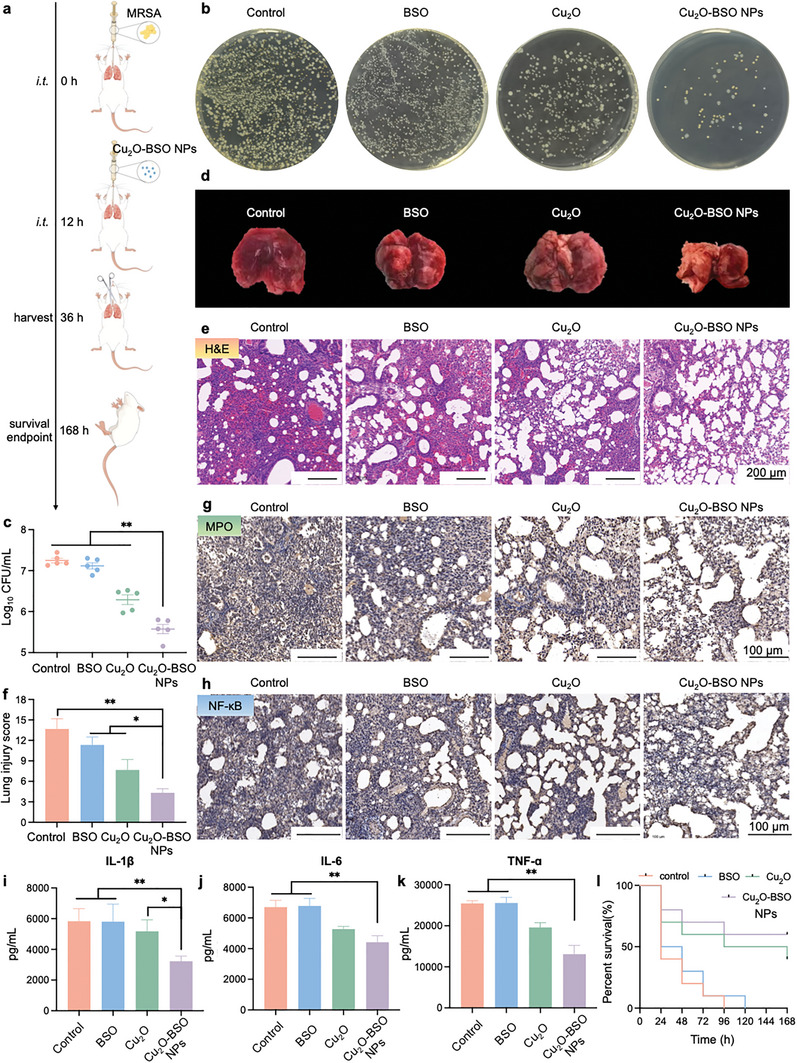
Antibacterial and anti‐inflammatory effects of Cu_2_O‐BSO NPs in vivo. a) Experimental diagram of Cu_2_O‐BSO NPs in the treatment of acute bacterial pneumonia. b,c) Agar plate photographs and quantitative analysis of MRSA after treatments of PBS, Cu_2_O, BSO, Cu_2_O‐BSO NPs at 36 h. d) Representative photographs of lungs harvested from treated mice after infection. e–h) H&E staining (e), quantitative analysis of H&E score (f), MPO staining (g) and NF‐κB staining (h) of lung tissue after infection. i–k) Quantitative analysis of the levels of IL‐ 1β, IL‐6, and TNF‐α in lung homogenates of different treatments after infection. l) Survival rate of MRSA pneumonia mice after corresponding treatment.

Encouraged by experiments in vitro, we further validated the antibacterial and anti‐inflammatory effects mediated by Cu_2_O‐BSO NPs in vivo. We first evaluated the toxicity and hemolysis of Cu_2_O‐BSO NPs in vitro before conducting in vivo activity studies. The results showed that Cu_2_O‐BSO NPs had good cell safety and blood compatibility (Figure S16, Supporting Information). After that, we established an acute pneumonia model by intratracheal injection of MRSA. Subsequently, we administered PBS, BSO, Cu_2_O and Cu_2_O‐BSO NPs via intratracheal injection, and lung tissues were harvested for subsequent experiments at 24 h post‐administration (**Figure** **9**a). The lung homogenates were subjected to SPM to assess the bacterial load within the lungs. The results pointed out that the Cu_2_O‐BSO NPs treatment group had the lowest CFU, representing the best antibacterial capability in vivo (Figure 9b,c).

After the antibacterial experiment, we examined the anti‐inflammatory effects of Cu_2_O‐BSO NPs to further evaluate its beneficial role in treating acute MRSA pneumonia. Similar to those treated with PBS, mice in the BSO group exhibited apparent pulmonary hemorrhage and edema; in contrast, the effects were less pronounced in the Cu_2_O and Cu_2_O‐BSO NPs groups, with the Cu_2_O‐BSO NPs group demonstrating the mildest symptoms (Figure 9d). Subsequent hematoxylin and eosin (H&E) analysis revealed alterations in the lung tissue structure of all groups, including congestion of alveolar spaces, interstitial edema, and inflammatory cell infiltration (Figure 9e). Notably, the alveolar tissue structure in the Cu_2_O‐BSO NPs treatment group remained relatively clear, with a small portion of edema and focal inflammatory cell infiltration in the alveolar cavities, resulting in an evidently less severe lung injury compared to other groups. The lung injury score pointed out that the PBS and BSO groups had the most severe injuries, followed by the Cu_2_O and Cu_2_O‐BSO NPs groups, and Cu_2_O‐BSO NPs groups with the least severe injuries (Figure 9f).

Myeloperoxidase (MPO) is a heme peroxidase predominantly localized in the azurophilic granules of neutrophils, which are released upon neutrophil activation, serving as a crucial antimicrobial defense mechanism.^[^ ^47^ ^]^ Variations in MPO levels and activity can therefore serve as indicators of neutrophil function and activation status, as well as provide insights into the extent of neutrophil infiltration in pulmonary tissues. Immunohistochemical (IHC) analysis revealed that the Cu_2_O‐BSO NPs treatment group exhibited the lowest expression of MPO, suggesting minimal neutrophil infiltration (Figure 9g; Figure S17, Supporting Information). Nuclear factor kappa‐B (NF‐κB), a protein complex implicated in cellular responses to various stimuli, including bacterial or viral antigens, plays a pivotal role in modulating the immune response to infections.^[^ ^48^ ^]^ Fluctuations in NF‐κB levels and activity may reflect the severity of lung tissue inflammation. Correspondingly, IHC analysis revealed that NF‐κB expression was apparently reduced in the Cu_2_O‐BSO NPs treatment group, demonstrating attenuated inflammation (Figure 9h; Figure S18, Supporting Information). Furthermore, the levels of pro‐inflammatory cytokines, such as IL‐1β, IL‐6 and TNF‐α, were assessed in lung tissues using ELISA. Consistently, the Cu_2_O‐BSO NPs group had the lowest production of pro‐inflammatory cytokines, demonstrating the lowest level of inflammation (Figure 9i–k).

Moreover, the survival curve demonstrated that all mice with acute MRSA pneumonia succumbed within 96 or 120 h following treatment with PBS or BSO, respectively (Figure 9l). Conversely, the survival rates rose 40% or 60% after treatment with Cu_2_O or Cu_2_O‐BSO NPs, respectively. Additionally, the Cu_2_O‐BSO NPs group maintained greater core body temperature than the control group, which experienced a drop to ≈30 °C by 24 h post‐infection (Figure S19, Supporting Information). Concurrently, compared to mice treated with PBS or BSO, those treated with Cu_2_O‐BSO NPs exhibited the lowest lung wet/dry weights at 36 h post‐infection, implying only mild edema (Figure S20, Supporting Information). Taken together, these results pointed out that the treatment with Cu_2_O‐BSO NPs can exert potent antibacterial and anti‐inflammatory effects, protecting mice from lung injury caused by excessive inflammatory responses, and ultimately improving the survival rate and prognosis of mice.

### 2.10 Preliminary Toxicity of Cu_2_O‐BSO NPs in vivo

**Figure 10:**
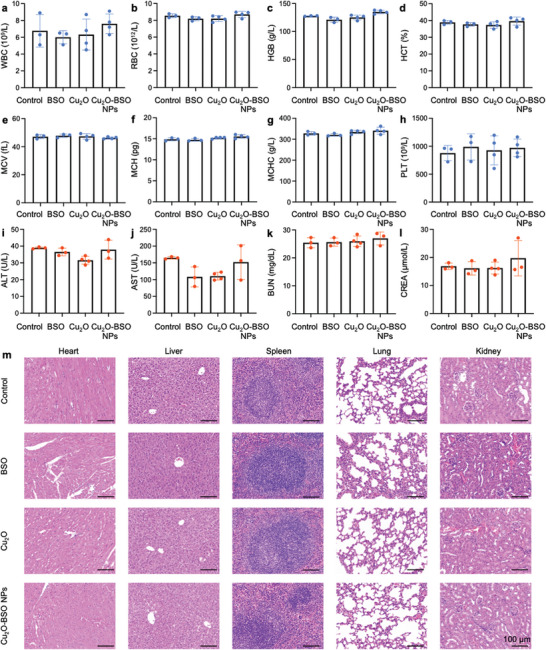
Preliminary toxicity of Cu_2_O, BSO, and Cu_2_O‐BSO NPs. a–l) Blood routine (WBC, white blood cell; RBC, red blood cell; HGB, hemoglobin; HCT, hematocrit; MCV, mean cell volume; MCH, mean corpuscular hemoglobin; MCHC, mean corpuscular hemoglobin concentration; PLT, platelet) and blood biochemistry analysis (ALT, alanine transferase; AST, aspartate transferase; BUN, blood urea nitrogen; CREA, creatinine) of healthy BALB/c mice after treatment Cu_2_O, BSO and Cu_2_O‐BSO NPs on day 14. m) H&E staining of heart, liver, spleen, lungs, and kidneys of healthy BALB/c mice in each group on day 14.

To further facilitate the advancement of our antimicrobial materials into clinical applications, it is imperative to conduct a thorough assessment of their toxicity. The long‐term effects of BSO, Cu_2_O, and Cu_2_O‐BSO NPs in healthy mice revealed that by day 14, both blood biochemical and routine blood indicators remained within normal parameters (**Figure** **10**a–l), and biological safety in vivo was demonstrated by the absence of inflammatory invasion within major organs (e.g., heart, liver, spleen, lungs and kidneys) (Figure 10m). Conclusively, all these results robustly confirmed their biocompatibility and hold promise for further clinical trials.

## 3 Conclusion

This study employed Cu_2_O as an inducer of cuproptosis‐like death and BSO as an amplifier of cuproptosis‐like death to prepare antimicrobial particles (Cu_2_O‐BSO NPs). Additionally, DOPA and PEG were utilized to enhance adhesion to the lung and penetration of the mucous membrane, respectively, providing a novel approach for treating MRSA‐induced acute pneumonia. in vitro, Cu_2_O‐BSO NPs exhibited outstanding broad‐spectrum antimicrobial and anti‐biofilm activity. By generating a high amount of ROS, it triggered lipid peroxidation, disrupted bacterial integrity, inhibited the respiratory chain and TCA cycle, suppressed QS system, reduced bacterial virulence, ultimately attributing to cuproptosis‐like death in bacteria. More importantly, Cu_2_O‐BSO NPs possessed great lung retention, excellent antibacterial activity in vivo, and can significantly enhance the survival rate of mice with acute bacterial pneumonia, reduce bacterial load in the lungs, and alleviate inflammatory damage. Furthermore, Cu_2_O‐BSO NPs can induce macrophages to eliminate bacteria through increased chemotaxis and phagocytosis. The pulmonary inhalation therapy utilizing Cu_2_O‐BSO NPs represents a non‐invasive drug delivery approach via the trachea and bronchial pathways. This method allows for precise regulation of pulmonary drug administration, achieving high bioavailability while minimizing systemic side effects. Consequently, it holds significant potential for the treatment of various pulmonary diseases. Beyond their application in the respiratory system, Cu_2_O‐BSO NPs exhibit broad‐spectrum antibacterial properties that can be effectively utilized in treating other drug‐resistant bacterial infections, including skin infections, osteomyelitis, and even sepsis, which has a significant potential for wide‐ranging clinical applications.

## 4 Experimental Section

### Synthesis of Cu_2_O‐BSO NPs

All chemicals and reagents were used as received without any further purification. Cu(NO_3_)_2_·3H_2_O, AA, and BSO were purchased from Aladdin (China). DOPA and PEG were obtained from Sigma–Aldrich (USA). Cu_2_O NPs were prepared by the reduction of Cu(NO_3_)_2_. In brief, 100 mg Cu(NO_3_)_2_·3H_2_O was dissolved in 90 mL ultra‐pure water, and then 10 ml AA (12 mg mL^−1^) and 2 mL NaOH (200 mg mL^−1^) were rapidly added at the same time. After the reaction for 30 min, Cu_2_O nanoparticles were obtained by centrifugation at 8000 rpm and re‐dispersed in 20 mL ultra‐pure water. Then, 100 mg DOPA and 20 mg BSO were added and incubated overnight. After centrifugation, 100 mg PEG was added and incubated for 3 h. Finally, Cu_2_O‐BSO NPs were obtained by centrifugation.

### Characterizations of Cu_2_O, Cu_2_O‐BSO NPs

The morphology of Cu_2_O‐BSO NPs was monitored with the SEM (HITACHI SU8010, Japan) and TEM (HITACHI HT7800, Japan). Optical absorption of Cu_2_O‐BSO NPs was measured on a UV–vis–NIR spectrophotometer (UV‐2600, Shimadzu, Japan). The dynamic light scattering and zeta potential were measured by DLS system (Zetasizer Nano‐ZSE, Malvern Instruments, UK). To determine the release capacity of the Cu_2_O‐BSO NPs in different pH environments (pH 6.0; pH 7.4). 0.5 mL 2.5 mg mL^−1^ Cu_2_O‐BSO NPs were placed in PBS of different pH (separated by 10 000 molecular weight dialysis bags), sampled at different time points (0.5, 1.5, 3.5, 5.5, 17.5, and 24 h), and the release amount of copper ions was detected by ICP‐MS.

### Lung Retention Capacity of Cu_2_O‐BSO NPs In Vivo

The Cu_2_O‐BSO NPs were coupled with IR783 to yield IR783‐Cu_2_O‐BSO NPs, 50 µL of which was slowly administered into the lungs of C57/BL mice by intratracheal injection.^[^ ^49^ ^]^ Afterward, the in vivo fluorescence signals were continuously observed in the mice at 6, 24, 48, and 96 h using the IVIS imaging system (Xenogen). Simultaneously, ex vivo fluorescence images were acquired from the heart, liver, spleen, lungs and kidneys of the mice at the same time points.

### Mucosal Penetration Ability of Cu_2_O‐BSO NPs

First, artificial airway mucus was prepared.^[^ ^50^ ^]^ Briefly, under magnetic stirring, dd H_2_O was used to dissolve mucin (from porcine stomach, type II) and sodium alginate (SA) overnight. On the day of the experiment, mucin solution (43.75 mg mL^−1^), calcium carbonate sodium salt solution (14 mg mL^−1^), d‐ (+) ‐gluconate delta‐lactone (GDL, 49.84 mg mL^−1^), and SA (21 mg mL^−1^) were mixed in a reaction flask under magnetic stirring for 1 min. Subsequently, a 0.1% gelatin solution was prepared, heated, and cooled to 50 °C into glass tubes (1 mL tube^−1^). After cooling and solidification, an additional 1 mL of artificial mucus was added to each tube. Then, 1 mL of Coomassie Brilliant Blue staining Cu_2_O NPs, DOPA‐Cu_2_O‐BSO and Cu_2_O‐BSO NPs were gently covered onto the artificial mucus, and the penetration of both in the mucus was observed at 0, 0.5, 1.5, 2, 3, and 4 h. To further quantify the permeability of the mucus, a transwell insertion method was developed.^[^ ^51^ ^]^ A transwell insert (Saining) with a pore size of 0.4 µm was placed on a 24‐well plate and covered with 100 mg of artificial mucus. The receptor chamber was filled with 500 µL of PBS. 100 µL of Cu_2_O NPs, DOPA‐Cu_2_O‐BSO and Cu_2_O‐BSO NPs were added to the donor chamber, respectively, and incubated in a 37 °C incubator. At different time points (0, 1, 1.5, 2, 2.5 h), 100 µL samples were collected from the receptor chamber, and the same amount of fresh PBS was added. All collected samples were tested for copper content using a Cu colorimetric assay kit (Elabscience).

### In Vitro Antibacterial Efficiency

BSO (250 µg mL^−1^), Cu_2_O (250 µg mL^−1^), and Cu_2_O‐BSO NPs (the concentration of Cu_2_O: 250 µg mL^−1^) were applied for following in vitro antibacterial experiments. The efficacy of BSO, Cu_2_O and Cu_2_O‐BSO NPs against MRSA (1 × 10^6^ CFU) and PAO1 (1 × 10^6^ CFU) was assessed by turbidimetric method and SPM.^[^ ^26^ ^]^ The fluorescence diagram of bacteria (1 × 10^8^ CFU) was conducted using SYTO 9 and PI dyes in CLSM (Zeiss LSM980).^[^ ^4a^ ^]^ Following this, MRSA and PAO1 from each group underwent fixation, dehydration, drying, slicing, staining, and were subsequently placed on a copper mesh for TEM imaging. In terms of evaluating the disruption of bacterial membrane integrity, the Bradford assay (Coomassie Brilliant Blue method) was employed to detect protein leakage in the bacterial supernatant. The optical density of 595 nm (OD 595) was measured by M5 microplate reader after corresponding operations according to the instructions. The depolarization of bacterial membrane potential was detected using the DiSC_3_(5) fluorescent probe, followed by recording of fluorescence intensity using ImageJ. The experiment of bacterial outer membrane permeability mainly uses NPN as a fluorescent probe to observe the integrity of bacterial outer membrane.

### In Vitro Anti‐Biofilm Capacity

The methodology employed in the anti‐biofilm experiment closely resembled previous research methods.^[^ ^26^, ^52^ ^]^ The concentration dependent anti‐biofilm experiments were verified by crystal violet, where the concentrations of Cu_2_O‐BSO NPs were 0, 125, 250 and 500 µg mL^−1^, respectively. BSO (500 µg mL^−1^), Cu_2_O (500 µg mL^−1^), and Cu_2_O‐BSO NPs (the concentration of Cu_2_O: 500 µg mL^−1^) were used in subsequent anti‐biofilm experiments. Specifically, in the biofilm destruction experiment, bacteria (1 × 10^7^ CFU) were introduced to a 24‐well plate and allowed to form a mature biofilm at 37 °C for 24 h. Subsequently, the supernatant was removed and replaced with blank TSB medium, TSB medium containing BSO, Cu_2_O and Cu_2_O‐BSO NPs, respectively, each of which was then incubated at 37 °C for an additional 24 h. Crystal violet staining and SPM were conducted after washing with PBS. The 3D image of the biofilms was acquired through the cultivation of biofilm on glasses, and then CLSM was used to obtain fluorescence image. In the biofilm inhibition experiment, bacteria were introduced into a 24‐well plate containing blank TSB medium, as well as TSB medium supplemented with BSO, Cu_2_O, and Cu_2_O‐BSO NPs. The subsequent procedures followed were identical to those of the biofilm destruction experiment. In the anti‐EPS experiment, Calcofluor white dye was used to stain each group.

### Cuproptosis‐Like Death Induced by Cu_2_O‐BSO NPs

The level of ROS in MRSA was assessed using the DCFH‐DA probe under CLSM. Additionally, the impact of BSO, Cu_2_O, and Cu_2_O‐BSO NPs on the bacterial respiratory chain complexes was investigated using bacterial respiratory chain complex I/II/III/IV assay (Chengong). The Cu content in bacteria was detected using a Cu detection kit (Elabscience). The pyruvate detection kit (Elabscience) assessed the accumulation of pyruvate in bacteria. The MDA detection kit (Solarbio) and BODIPY581/591‐C11 fluorescent probe (Thermo Fisher) were used to evaluate the level of lipid peroxidation in bacteria. GSH and GSSG detection kits (Beyotime) were utilized to measure the ratio of GSH and GSSG. The DNA fragmentation of bacteria was verified using a DNA damage detection kit (γ‐H2AX immunofluorescence method, Beyotime). To further investigate the impact of cuproptosis‐based death inhibitors on antibacterial activity, blank TSB and EDTA were co‐incubated with MRSA, respectively. After 24 h of incubation at 37 °C, bacterial viability was determined by SPM.

### Treatment of MRSA‐Induced Acute Pneumonia

First, a mouse model for acute MRSA pneumonia was established. Specifically, the mice were anesthetized by tribromoethanol (Meilun), and 50 µL of PBS‐suspended MRSA (5 × 10^7^ CFU) was slowly injected into the trachea of the mice with the aid of a laryngoscope through an injector equipped with a tracheal cannula.^[^ ^53^ ^]^ The airways of the mice were then injected with 50 µL of PBS, BSO (5 mg kg^−1^), Cu_2_O (5 mg kg^−1^), and Cu_2_O‐BSO NPs (the concentration of Cu_2_O: 5 mg kg^−1^) 12 h after the pneumonia model had been established. The mice were subsequently euthanized at 36 h, and their lungs were surgically removed. To compare the survival rates and core body temperatures of the mice in different groups, MRSA (2×10^8^ CFU) was administered intratracheally to each mouse (*n* = 10/group).

### Antibacterial and Anti‐Inflammatory Effects In Vivo

Following the removal and photographic documentation of the lungs, they were subjected to homogenization using 500 µL of PBS and sterile grinding beads in a Tissue Prep Tissue Lyser to yield lung homogenates. The in vivo antibacterial efficacy of the respective treatments was subsequently evaluated in the lung homogenates utilizing SPM (*n* = 5/group). The levels of pro‐inflammatory cytokines IL‐6, IL‐1β, and TNF‐α were determined through ELISA kits (Elabscience) to confirm their anti‐inflammatory properties (*n* = 3/group). H&E staining was employed for the visualization of inflammatory cell infiltration in the lung tissue, while immunohistochemical staining was utilized to observe alterations in MPO and NF‐κB (*n* = 3/group).

### Immunomodulatory Experiments In Vitro

The MH‐S were seeded into 6‐well plates (Saining) at a density of 2 × 10^5^ cells well^−1^. After culturing for 1 day at 37 °C, the culture medium was replaced with fresh MH‐S cell complete culture medium (Pricella) containing BSO, Cu_2_O, and Cu_2_O‐BSO NPs. After incubating for 2 days at 37 °C, the supernatant of the culture medium was collected and the concentrations of TNF‐α, IL‐6, and IL‐1β were detected using ELISA kits. The remaining cells were subjected to flow cytometry analysis. Each group of cells was centrifuged, washed, and resuspended in 500 µL of PBS, which contained an anti‐mouse CD86 antibody coupled with FITC (Elabscience) and an anti‐mouse CD206 antibody coupled with PE (Elabscience). Then, they were incubated at 4 °C in the dark for 30 min. Eventually, the expression of CD86 and CD206 on the surface of the MH‐S cells was detected via a flow cytometer (BD FACSCanto II).

### Functional Evaluation of Macrophages

The MH‐S cells were introduced into a confocal dish and incubated at 37 °C for 24 h. After that, PBS, BSO, Cu_2_O, and Cu_2_O‐BSO NPs were added, respectively, and the cells were incubated for an additional 48 h. Following the addition of GFP‐labeled MRSA (MRSA: cell = 10–20:1), the incubation was continued at 37 °C for 2 h. The dishes were then washed three times with PBS and replenished with fresh medium containing 200 µg mL^−1^ gentamicin to eliminate extracellular MRSA. After three PBS washed, 1% Triton X‐100 was added to each well to permeabilize the macrophage cell membrane and induce phagocytic bacterial leakage. CFU counts were then performed using a gradient dilution method and SPM. For another, after gentamicin treatment, the medium was discarded, the cells were washed, fixed, and permeabilized with Triton X‐100, and then TRITC phalloidin (Yeasen) and DAPI (Beyotime) staining were applied, followed by capturing all cell images using a CLSM (Zeiss LSM980).

### Statistical Analysis

All data were expressed as mean ± standard mean error. Student's *t*‐test or a one‐way analysis (ANOVA) of variance were used to compare the differences between two or more groups, respectively. Tukey test was used for pairwise comparison in ANOVA. The in vitro experiment was repeated 3 times, with 3–5 animals per group in vivo to reduce error. ^*^ *p* < 0.05 and ^**^ *p* < 0.01 were considered statistically significant differences. All statistics were analyzed using GraphPad Prism10 software.

## Conflict of Interest

The authors declare no conflict of interest.

## Author Contributions

H.H and S.H. contributed equally to this work. Z.M. and X.F. conceived the idea, designed the project, provided supervision and guidance on experimental planning and funding. H.H.Q. and H.S.Y. overall methodology, experiments, results and discussion, data analysis, and the first draft of the manuscript and revision. L.F., Z.W.T., and Z.Z.W. assisted in animal experiments. C.J.R., L.X.Y. and X.J.Y. provide technical support.

## Supporting information



Supporting Information

## Data Availability

The data that support the findings of this study are available from the corresponding author upon reasonable request.
