# Supplementary material for: Mucous Permeable Nanoparticle for Inducing Cuproptosis‐Like Death In Broad‐Spectrum Bacteria for Nebulized Treatment of Acute Pneumonia
Source: Adv Sci (Weinh). 2025 Feb 22;12(15):2408580. doi: 10.1002/advs.202408580 (PMC12005761; doi:10.1002/advs.202408580)
Supplement: Supplementary file 1 — Supporting Information [file ADVS-12-2408580-s001.docx]

**Mucous permeable nanoparticle for inducing cuproptosis-like death in broad-spectrum bacteria for nebulized treatment of acute pneumonia**

Huiqun Hu ^a, #^, Shiyuan Hua ^b, c, #^, Feng Lu ^d^, Wenting Zhang ^a^, Zengwen Zhang ^a^, Jiarong Cui ^b, c^, Xiaoyue Lei ^e^, Jingyan Xia ^f^，Feng Xu ^a, g,^ * Min Zhou ^a, b, c, h, i,^ *

a Department of Infectious Diseases, the Second Affiliated Hospital, Zhejiang University School of Medicine, Hangzhou, 310009, China.

b Institute of Translational Medicine, Zhejiang University, Hangzhou, 310029, China.

c Zhejiang University-University of Edinburgh Institute (ZJU-UoE Institute), Zhejiang University School of Medicine, Zhejiang University, Haining 314400, China

d Department of Orthopedics, The Affiliated Changzhou No.2 People's Hospital of Nanjing Medical University, Changzhou, 213003, China.

e The Affiliated Hospital of Stomatology, School of Stomatology, Zhejiang University School of Medicine, and Key Laboratory of Oral Biomedical Research of Zhejiang Province, Hangzhou, Zhejiang,310006, China.

f Department of Radiation Therapy, the Second Affiliated Hospital, Zhejiang University School of Medicine, Hangzhou, Zhejiang 310009, P.R. China.

g Research Center for Life Science and Human Health, Binjiang Institute of Zhejiang University, Hangzhou 310053, China.

h State Key Laboratory of Modern Optical Instrumentations, Zhejiang University,

Hangzhou 310058, China

# These authors contributed equally.

Correspondence and requests for materials should be addressed to

M.Z. (email: zhoum@zju.edu.cn),

or to F.X. (email: [xufeng99@zju.edu.cn](mailto:xufeng99@zju.edu.cn))

**Materials and instruments**

Methicillin-resistant *Staphylococcus aureus* (MRSA, ATCC 43300) and pseudomonas aeruginosa (PAO1, ATCC 15692) were obtained from the American Type Culture Collection, respectively. Soybean–Casein Digest Broth (TSB) broth, TSB broth agar, Luria-Bertani (LB) broth, LB broth agar were purchased from Meilun (Dalian, China). Dulbecco's Modified Eagle Medium (DMEM) was obtained from Gibco (USA), MH-S cell specific medium was purchased from Pricella (Wuhan, China). Double distilled water (ddH_2_O) was obtained from the Milli-Q purification system.

**Bacterial Culture**

MRSA was aerobically cultured in TSB broth (shaking at 250 rpm) or TSB broth agar plates overnight at 37 °C. The MRSA expressing green fluorescent protein (GFP) and luciferase (MRSA-GFP-Luci) was constructed by Forhigh Biotech Co., Ltd, and incubated in TSBcm (TSB with 1 μg/mL chloramphenicol) or TSBcm agar (TSB agar with 1 μg/mL chloramphenicol) plates. PAO1 was aerobically cultured in LB broth (shaking at 250 rpm) or LB broth agar plates overnight at 37 °C.

**MIC of Cu_2_O-BSO NPs**

The minimum inhibitory concentration (MIC) was determined using a standard microdilution method to evaluate the antibacterial activity of Cu_2_O-BSO NPs. First, Cu_2_O-BSO NPs (0, 7.82, 15.63, 31.25, 62.5, 125, 250, 500 µg/mL) were prepared in TSB as two time-series dilutions for further processing. These different concentrations of diluted Cu_2_O-BSO NPs solutions were added to a 96-well plate at a rate of 50 μL/well. Then, the bacterial suspension diluted to 1×10^6^ CFU/mL was inoculated into each well (50 μL/well). After incubation at 37°C for 24 h, the OD600 of each well was measured and the survival rate of MRSA was calculated. The MIC is defined as the corresponding minimum concentration that results in a bacterial survival rate of less than 10%. The MIC of PAO1 is similar to that of MRSA.

**Animals**

Female BALB/c mice (6-8 weeks old) were purchased from Shanghai SLAC Laboratory Animal Co., Ltd. All mouse operations were approved by the Institutional Animal Care and Use Committee of the Second Affiliated Hospital of Zhejiang University School of Medicine, and were conducted in accordance with the ethical guidelines of the National Institutes of Health. When determining the sample size for animal experiments, specific statistical factors were not considered.

**Blood compatibility**

2% rabbit red blood cell suspensions were prepared and respectively mixed with BSO (250 µg/mL), Cu_2_O (250 µg/mL), and Cu_2_O-BSO NPs (250 µg/mL) at 37 °C for 1 h (v:v=1:1). Then these complexes were centrifuged at 2400 rpm for 5 min (PBS-treated was a negative control while ddH_2_O-treated was a positive control). Finally, the OD 540 values of the supernatants were measured to calculate the hemolysis percentage. Hemolysis rate less than 5% is considered to have good blood compatibility.

Hemolysis rate (%) = (OD _sample_ – OD _negative_) / (OD _positive_ – OD _negative_) × 100%

**Cytotoxicity**

Human bronchial epithelioid cells (BEAS-2B) were derived from the US type culture collection (ATCC CRL-9609). Then 100 μL of cell suspension was seeded in a 96-well plate (5x10^4^ cells /mL), and cultured until the cell density reached 80%-90%. The original medium was then replaced with a fresh Cu_2_O-BSO NPs -containing (0, 31.25, 62.5, 125, 250 µg/mL) medium for 24 h. Finally, the activity of cell was detected with CCK-8 kit.

As for cell live/dead staining, 1 mL of cell suspension was inoculated on a 24-well plate (5x10^4^ cells /mL), and cultured until the cell density reached 80%-90%. The original medium was then replaced with a fresh medium containing BSO (250 µg/mL), Cu_2_O (250 µg/mL), and Cu_2_O-BSO NPs (250 µg/mL), respectively. After incubation for 24 h, the cell survival rate was measured by Calcein-AM/PI staining kit (Yeasen, Shanghai, China) under confocal laser scanning microscope (CLSM).

***In vivo* toxicity test**

14 days after intratracheal instillation of BSO, Cu_2_O, Cu_2_O-BSO NPs in BALB/c mice, blood biochemical and routine tests were performed. The heart, lungs, liver, spleen, and kidneys of mice were collected, fixed with 10% formalin, routinely dehydrated, embedded in paraffin, and sliced to a thickness of 5 μm. The sections were stained with H&E for toxicological and histological examination.

**Anti-virulence assays**

Briefly, after treatment with blank TSB, BSO, Cu_2_O, Cu_2_O-BSO NPs, the activity of virulence factors was determined by bacterial culture supernatant. Firstly, the supernatants of different bacteria were incubated with 2% rabbit red blood cell suspensions at 37 °C for 1 h, And then the OD 540 values of the supernatants were measured to evaluate the degree of hemolysis.

**Figure S1.** Size distribution plot of Cu_2_O-BSO NPs before and after PEG modification.

**
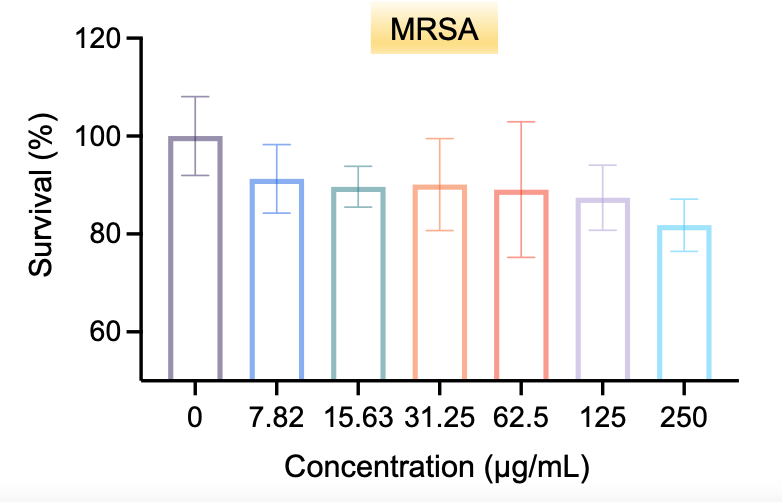
**

**Figure S2.** The anti-MRSA activity of ampicillin.

**
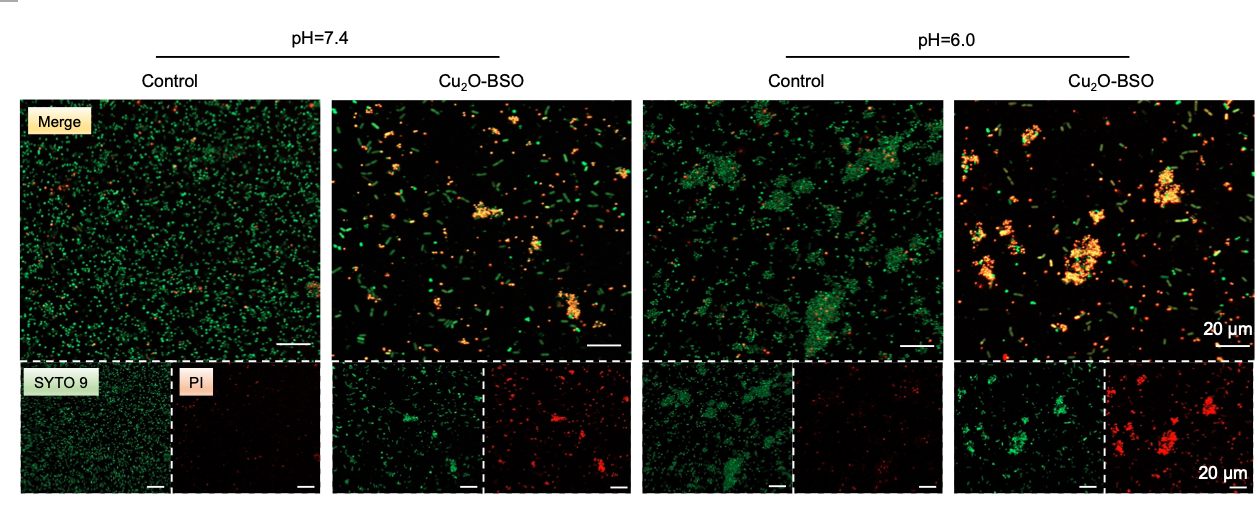
**

**Figure S3.** Live/dead staining images of MRSA processed by blank TSB and Cu_2_O-BSO NPs at pH 6 and pH 7.4.

**
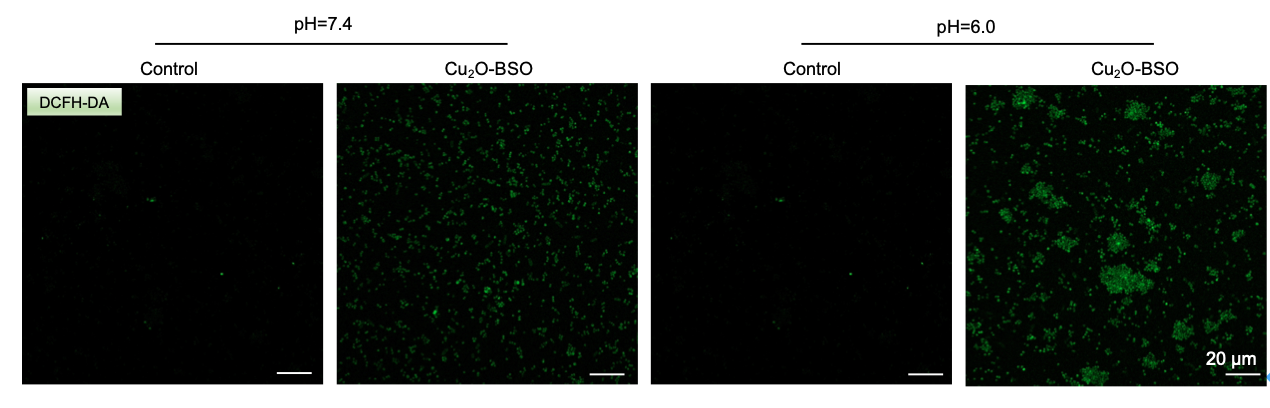
**

**Figure S4.** DCFH-DA staining images of MRSA processed by blank TSB and Cu_2_O-BSO NPs at pH 6 and pH 7.4.

**
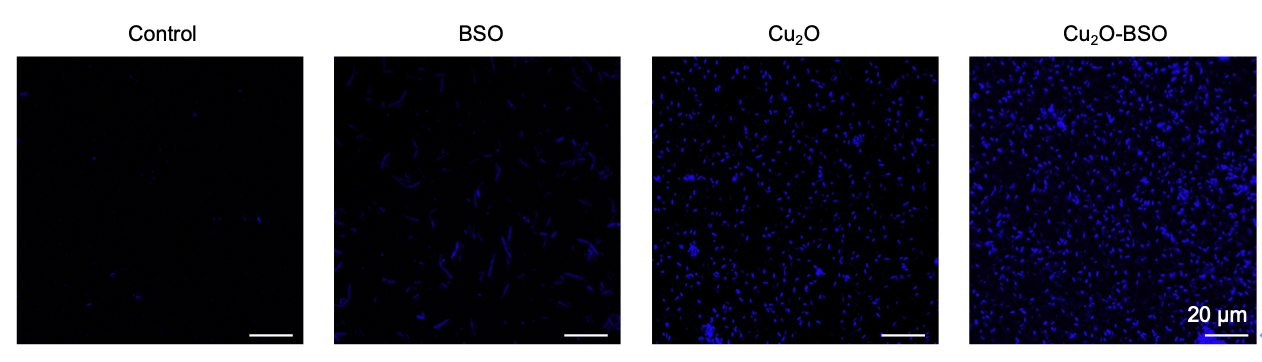
**

**Figure S5.** Fluorescence image of bacterial outer membrane permeability by NPN method.

**
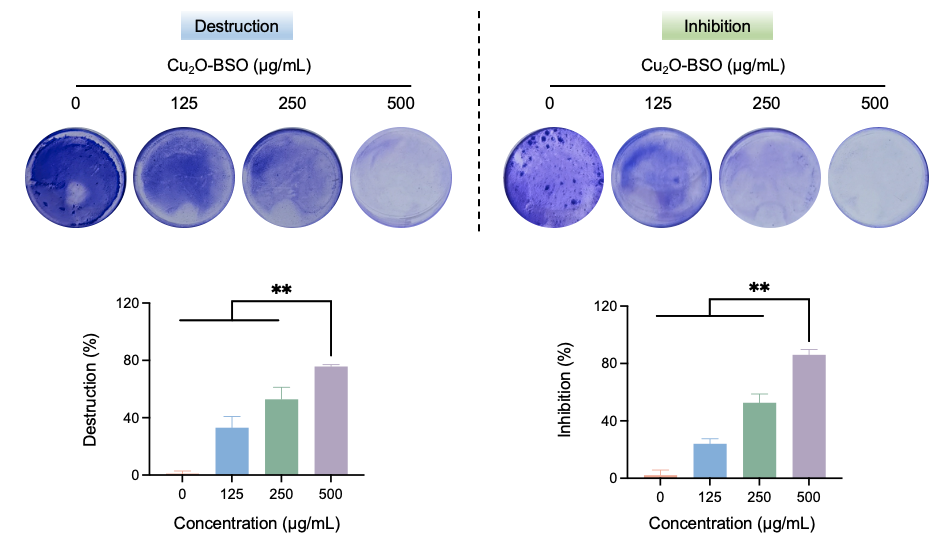
**

**Figure S6.** The anti-biofilm ability of different concentrations of Cu_2_O-BSO NPs.

**
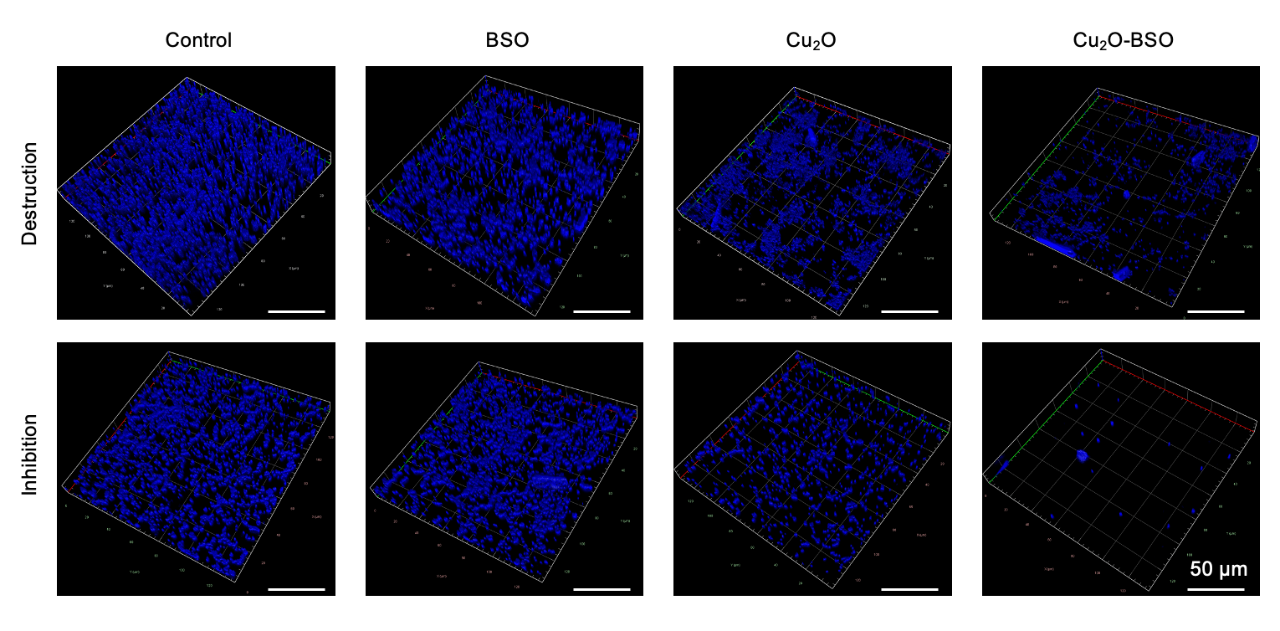
**

**Figure S7.** 3D images of EPS after treated with blank TSB, BSO, Cu_2_O, Cu_2_O-BSO NPs.

**Figure S8.** Scatter plot of expression difference.

**
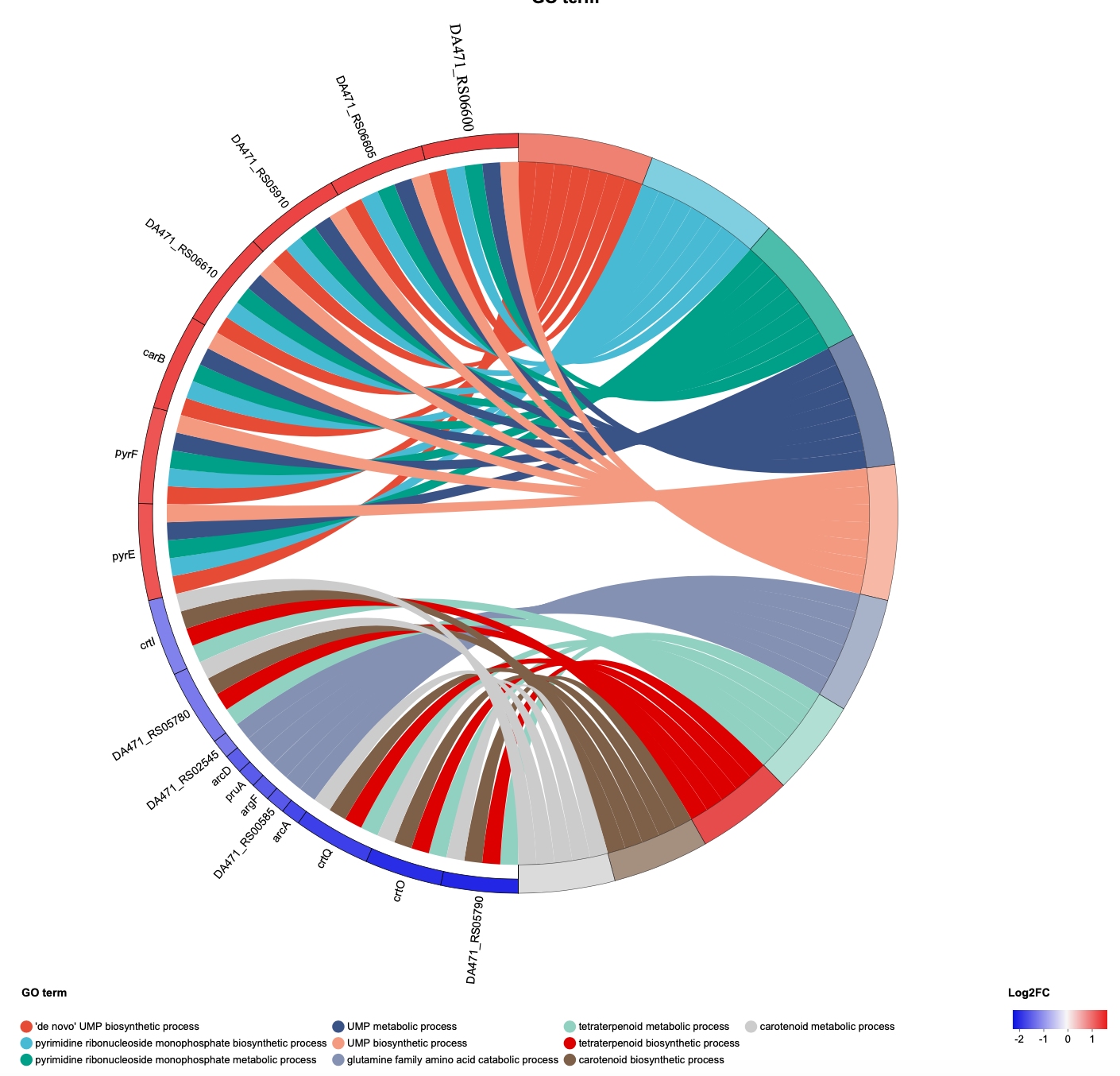
**

**Figure S9.** Chordal graph of GO enrichment.

**
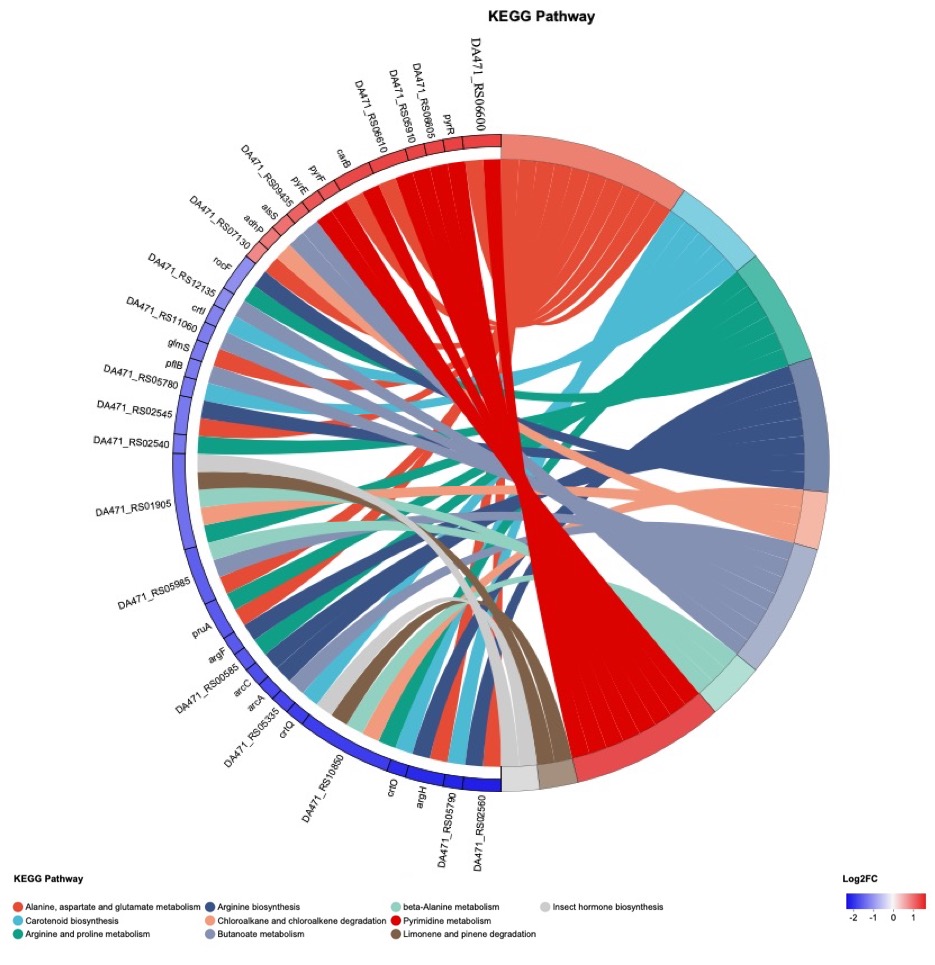
**

**Figure S10.** Chordal graph of KEGG enrichment.

**Figure S11.** Heat map of genes associated with *staphylococcus aureus* infection.


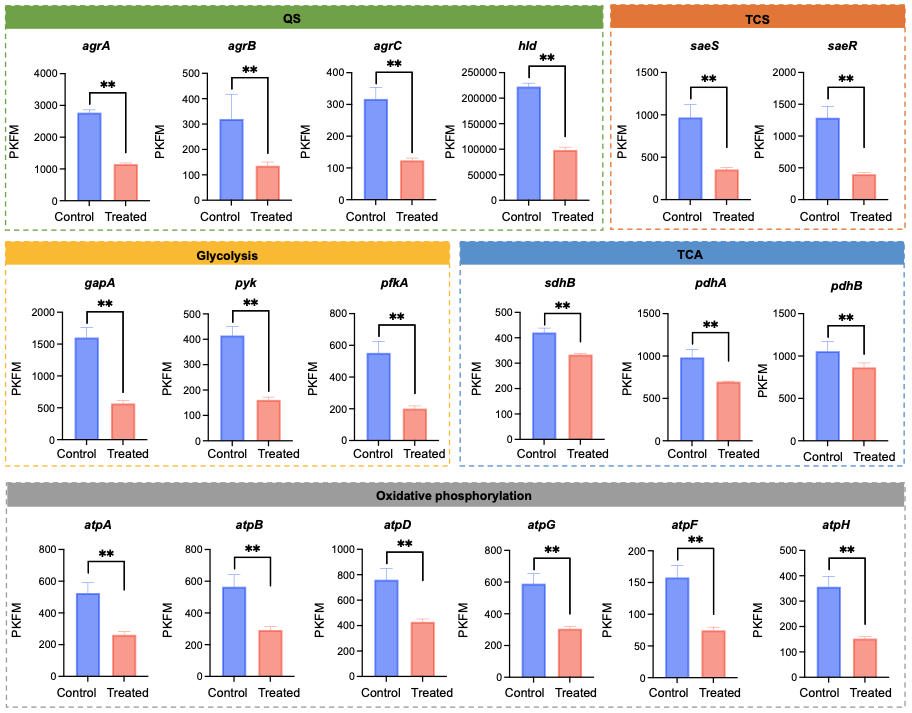


**Figure S12.** Fragments per kilobase milliom (FPKM) of *agrA, agrB, agrC, hld, saeR, saeS, gapA, pyk, pfkA, sdhB, pdhA, pdhB and atpA/B/D/G/F/H* of MRSA.

**Figure S13.** Photographs and quantitative analysis of the hemolytic reaction after different treatments.


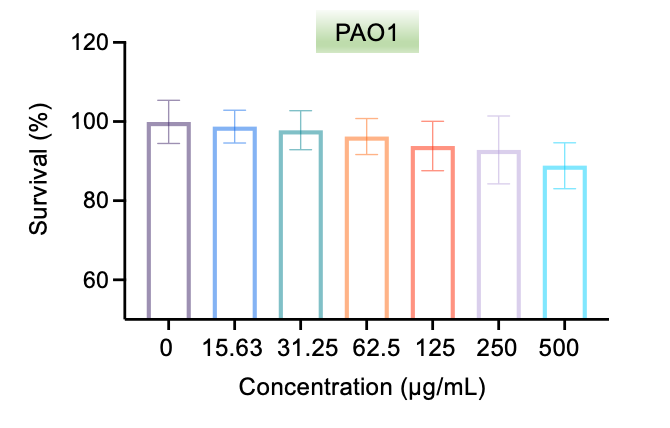


**Figure S14.** The anti-PAO1 activity of ampicillin.

**
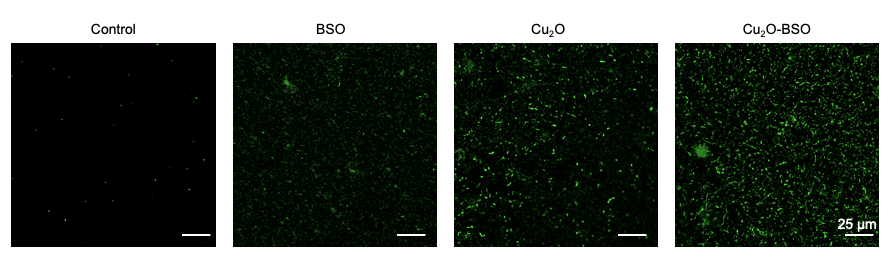
**

**Figure S15.** ROS level of the MH-S cells after different treatments.


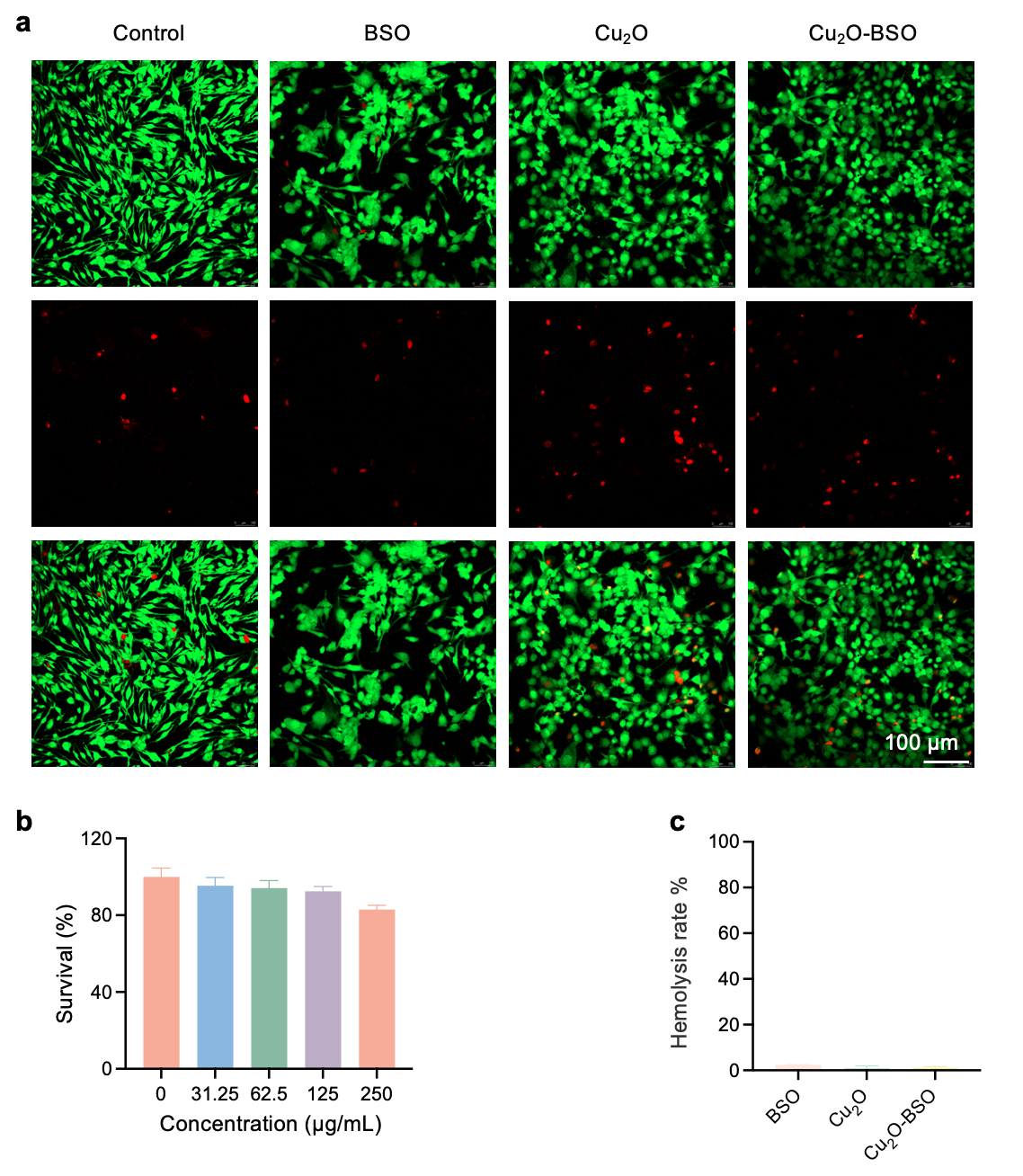


**Figure S16.** (a) Live/dead staining images of BEAS-2B treated with blank TSB, BSO, Cu_2_O and Cu_2_O-BSO NPs. (b) Survival of BEAS-2B treated with different concentrations of Cu_2_O-BSO NPs. (c) Hemolysis rate of BEAS-2B treated with BSO, Cu_2_O and Cu_2_O-BSO NPs.

**Figure S17.** Quantitative analysis of MPO area ratio in different groups

**Figure S18.** Quantitative analysis of NF-κB levels in different groups.

**
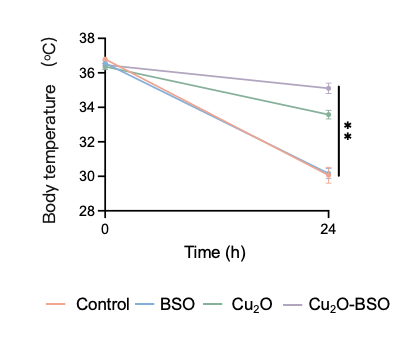
**

**Figure S19.** Body temperature of BALB/c in different groups.

**Figure S20.** Wet weight/dry weight ratio of lungs harvested in different groups.
